# Habitat Quality Differentiation and Consequences for Ecosystem Service Provision of an Amazonian Hyperdominant Tree Species

**DOI:** 10.3389/fpls.2021.621064

**Published:** 2021-03-31

**Authors:** Evert Thomas, Merel Jansen, Fidel Chiriboga-Arroyo, Lúcia H. O. Wadt, Ronald Corvera-Gomringer, Rachel Judith Atkinson, Stephen P. Bonser, Manuel Gabriel Velasquez-Ramirez, Brenton Ladd

**Affiliations:** ^1^Bioversity International, Lima, Peru; ^2^Department of Environmental Systems Science, Institute of Terrestrial Ecosystems, Ecosystem Management, ETH Zürich, Zurich, Switzerland; ^3^Center for International Forestry Research -CIFOR, Lima, Peru; ^4^Embrapa Rondônia, Porto Velho, Brazil; ^5^Instituto de Investigaciones de la Amazonia Peruana – IIAP, Puerto Maldonado, Peru; ^6^School of Biological, Earth and Environmental Science, Ecology & Evolution Research Centre, University of New South Wales, Sydney, NSW, Australia; ^7^Escuela de Agroforestería, Universidad Científica del Sur, Lima, Peru

**Keywords:** spatial aggregation, *Bertholletia excelsa*, carbon sequestration, growth differentiation balance framework, Janzen–Connell hypothesis, negative density dependence (NDD), ecosystem service trade-offs, environmental gradients

## Abstract

Ecosystem services of Amazonian forests are disproportionally produced by a limited set of hyperdominant tree species. Yet the spatial variation in the delivery of ecosystem services by individual hyperdominant species across their distribution ranges and corresponding environmental gradients is poorly understood. Here, we use the concept of habitat quality to unravel the effect of environmental gradients on seed production and aboveground biomass (AGB) of the Brazil nut, one of Amazonia’s largest and most long-lived hyperdominants. We find that a range of climate and soil gradients create trade-offs between density and fitness of Brazil nut trees. Density responses to environmental gradients were in line with predictions under the Janzen–Connell and Herms–Mattson hypotheses, whereas tree fitness responses were in line with resource requirements of trees over their life cycle. These trade-offs resulted in divergent responses in area-based seed production and AGB. While seed production and AGB of individual trees (i.e., fitness) responded similarly to most environmental gradients, they showed opposite tendencies to tree density for almost half of the gradients. However, for gradients creating opposite fitness-density responses, area-based seed production was invariable, while trends in area-based AGB tended to mirror the response of tree density. We conclude that while the relation between environmental gradients and tree density is generally indicative of the response of AGB accumulation in a given area of forest, this is not necessarily the case for fruit production.

## Introduction

Amazonian rainforests generate vital ecosystem services (Strand et al., 2018). They harbor the largest plant biodiversity on Earth (ter Steege et al., 2013), represent the biggest pool of tropical carbon (Brienen et al., 2015), and are an important source of timber (Rutishauser et al., 2015) and non-timber products (Shackleton et al., 2011). While the remarkable diversity of these forests undoubtedly plays a role in the generation of ecosystem services (Cardinale et al., 2011; Poorter et al., 2015; Liang et al., 2016), there is increasing evidence that some services, such as wood production and carbon storage, are disproportionally produced by a small number of hyperdominant tree species that are extremely common and abundant in one or more Amazonian forest regions (ter Steege et al., 2013; Fauset et al., 2015). However, the spatial variation in the delivery of ecosystem services by individual hyperdominant species across their distribution ranges and corresponding environmental gradients remains understudied.

**FIGURE 1:**
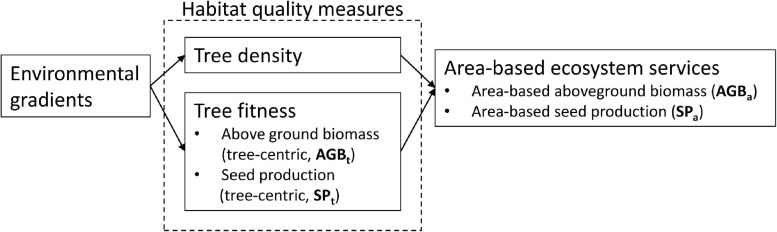
Conceptual representation of the relation between environmental gradients, habitat quality measures, and area-based ecosystem services.

The amount of ecosystem services generated in a given area of forest by a tree species is governed by the combination of the number of individual trees in that area (population density) (Winfree et al., 2015; Gaston et al., 2018) and the state and performance of the individual trees such as tree growth, survival, and reproduction (Figure 1). The reproductive success of tropical trees is vital for maintaining healthy recruitment levels and supporting forest food chains (Staggemeier et al., 2017), and also for the sustainability of non-timber forest product (NTFP) extractive economies. Tree survival over time and growth rate are generally associated with tree size (Rozendaal and Zuidema, 2011), and hence sequestration of carbon, a key ecosystem service for climate regulation and wood production. Therefore, to understand the effect of spatial variation on the delivery of ecosystem services by a tree species, one must understand how tree density and individual tree performance vary along environmental gradients.

The concept of habitat quality offers a powerful framework to analyze this. Habitat quality is a measure of the probability that a particular habitat allows for the long-term persistence of a local population (Johnson, 2007; Oliver et al., 2012), with the key indicators of habitat quality for a given species being population density (Pimm et al., 1988; Gutiérrez et al., 2013) and fitness. Fitness can be measured as an individual’s contribution to population persistence and is the combination of tree performance in terms of reproductive success (fertility), growth (Jansen et al., 2012), and survival (Bradshaw and McMahon, 2008; Bean et al., 2014). The ecosystem services that a tree species provides in a certain area thus are to a large extent dependent on the habitat quality of the area. However, within habitat quality there may be differentiation; i.e., tree density and fitness may be negatively correlated, and different fitness measures may not all be positively correlated among each other. Such differentiation of habitat quality is well-documented in animal species (Johnson, 2007). For example, in bird species, increasing population density has been shown to diminish the average fecundity of breeding pairs (Rodenhouse et al., 2003), but in tropical tree species, this mechanism remains understudied.

An important indication of the differentiation of density- and fitness-related measures in tropical tree species is the pervasive manifestation of conspecific negative distance or density-dependent (CNDDD) effects on the recruitment (Harms et al., 2000; Comita et al., 2014; LaManna et al., 2017), survival (He and Duncan, 2000; Hubbell et al., 2001; Zhang et al., 2009), growth (Uriarte et al., 2004), and fruit production (Alvarez-Buylla, 1994; Jones and Comita, 2008; Thomas et al., 2017, 2018) of tree species around the world. CNDDD effects on tree recruitment are explained by the Janzen (1970)–Connell (1971) hypothesis, which predicts that host-specific seed predators, herbivores, and pathogens are attracted to seeds, seedlings, and juvenile plants at high density or close to adult conspecifics, resulting in a higher mortality compared with other situations. CNDDD effects on the fitness of adult trees, on the other hand, have been related to intraspecific competition for resources and fine-scale genetic structuring (Hubbell et al., 2001; Jones and Comita, 2008; Thomas et al., 2018).

Furthermore, habitat quality measures at the level of individuals (tree-centric) and populations (area-based) are expected to show idiosyncratic responses to environmental gradients that exist across tree species distribution ranges. For example, individual tree growth and reproductive success have been found to respond differently to environmental variables in Neotropical tree species in accordance with different resource requirements and environmental tolerance limits throughout the trees’ life cycles (Staudhammer et al., 2013; Alfaro-Sánchez et al., 2017). Variation in conspecific tree density and aggregation across environmental gradients, on the other hand, is partly related to environmentally controlled CNDDD effects of recruitment (Bachelot et al., 2016; LaManna et al., 2016, 2017). High rainfall and low seasonality in the tropics favor desiccation-intolerant insects and fungi that are directly responsible for promoting high rates of CNDDD plant mortality (Janzen, 1970; Connell, 1971). On the other hand, infertile soils or unfavorable rooting conditions tend to favor greater allocation to anti-herbivore defenses in trees, which leads to lower rates of pest- and disease-related mortality (Givnish, 1999). This is explained by the hypothesis of Herms and Mattson (1992), which postulates that plant defense is premised upon a physiological trade-off between growth (cell division and enlargement) and differentiation (chemical and morphological changes leading to cell maturation and specialization, including synthesis of defense compounds), referred to as the g*rowth differentiation balance framework*. Nutrient and light-rich conditions favor growth at the expense of defense compounds to enhance a plant’s competitive advantage. By contrast, in resource-limited environments, several factors interact to favor the selection of high levels of secondary metabolism (Herms and Mattson, 1992). In accordance with the Janzen–Connell and Herms–Mattson hypotheses, precipitation (Comita et al., 2014) and soil fertility (LaManna et al., 2016) have been found to positively influence CNDDD effects on tree recruitment and hence lower conspecific tree density and aggregation (Comita et al., 2010; Mangan et al., 2010).

The question remains as to how different responses to environmental gradients of area-based (density) and tree-centric (fitness) habitat quality indicators might influence the potential provision of ecosystem services by a tree species in a given area of forest. In this study, we test two hypotheses. The first hypothesis is that the relationships between environmental gradients and ecosystem service generation of a tree species in a given area of forest mirror those of its density, regardless of possible divergent responses of fitness variables (cf. Winfree et al., 2015; Gaston et al., 2018). The second hypothesis we test is that conspecific density responds to environmental gradients in line with the predictions under the Janzen–Connell and Herms–Mattson hypotheses, while fitness variables respond to environmental gradients in line with niche preferences and resource requirements throughout a tree’s life cycle. More specifically, we expect environmental conditions that favor pests and diseases (higher precipitation and air temperatures and lower seasonality) and disfavor plant defense mechanisms against these (higher soil fertility and lower soil density) to be associated with lower tree densities. By contrast, higher soil fertility and precipitation are expected to positively influence seed production and biomass accumulation of individual trees, while higher air temperature is anticipated to have a negative influence on these variables in line with findings for tree species around the world (Feeley et al., 2007; Clark et al., 2010, 2016; Pérez-Ramos et al., 2010; Rozendaal and Zuidema, 2011; Girard et al., 2012; Wagner et al., 2014; Alfaro-Sánchez et al., 2017).

We test these hypotheses for the Brazil nut tree (*Bertholletia excelsa*, an Amazonian hyperdominant), using the fitness measures seed production and aboveground biomass (AGB). As the estimated seed production and AGB of focal Brazil nut trees have been found to be negatively influenced by both the density and spatial aggregation of their conspecific neighborhoods, which are not linearly correlated (Thomas et al., 2018), we additionally assessed the relation between spatial aggregation and environmental variables. Spatial aggregation is not considered as a measure of habitat quality, but if environmental gradients on Brazil nut tree density reflect CNDDD effects, spatial aggregation is expected to show congruent trends.

## Materials and Methods

### Study Species

The Brazil nut tree is an emergent of up to 60 m tall and one of the largest and most long-lived of all hyperdominant tree species in Amazonia (Vieira et al., 2005; ter Steege et al., 2013; Schöngart et al., 2015). Brazil nut trees not only play a keystone role in the ecology and nutrient cycling of Amazonian forests (Wadt et al., 2005) but have also supported human livelihoods since the peopling of the Amazon basin (Shepard and Ramirez, 2011; Thomas et al., 2015). Brazil nut seed remains a cornerstone NTFP in Amazonia, and the species plays a pivotal role in carbon sequestration (Fauset et al., 2015; Selaya et al., 2017). Brazil nut has an aggregated distribution pattern due to the combined effects of short-distance seed dispersal by rodents (Haugaasen et al., 2010) and anthropogenic activities (Shepard and Ramirez, 2011; Thomas et al., 2014, 2015). However, different lines of evidence suggest that in Peru the effect of anthropogenic activities appears to have been minimal as compared with central and eastern Amazonia (Thomas et al., 2015; Rockwell et al., 2017; Porcher et al., 2018). Also, human harvesting of Brazil nut seeds is unlikely to negatively impact regeneration. A study in nearby Acre, Brazil, tracked the fate of almost 7,000 Brazil nut fruits right after falling on the ground and concluded that Brazil nut harvesting is unlikely to threaten recruitment (de Wadt et al., 2018). The main reason for this is that (1) harvesters typically enter the forest only after most fruits have fallen (over a 3-month period) because of the big risk of being injured (or killed) by falling fruits and (2) the main dispersers (agoutis) typically open fruits within days after falling.

**FIGURE 2:**
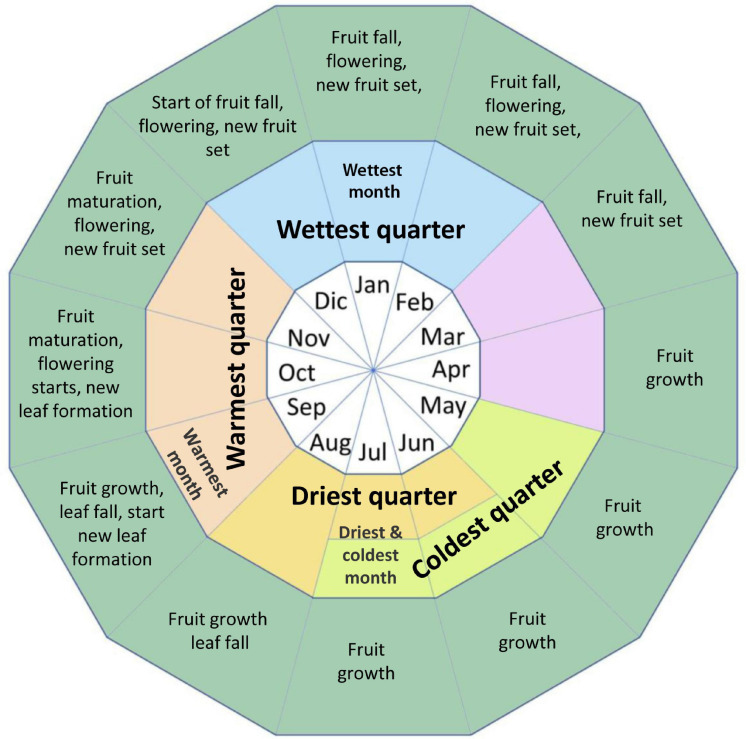
Phenology of the Brazil nut in the study regions based on Corvera-Gomringer et al. (2010) and the authors’ personal long-term observations.

Figure 2 shows the phenology of the Brazil nut in the study regions based on Corvera-Gomringer et al. (2010) and the authors’ personal long-term observations. As the fruits take 12–14 months to mature, there are several critical phases throughout the year where climate variables may influence flowering, fruit set, and fruit growth and development. Furthermore, the Brazil nut has a semi-deciduous behavior in the study region, and the species typically starts shedding leaves at the end of the dry season, and new leaf growth occurs throughout the warmest quarter of the year, suggesting that precipitation and air temperature in these periods of the year might influence biomass accumulation.

### Data

**FIGURE 3:**
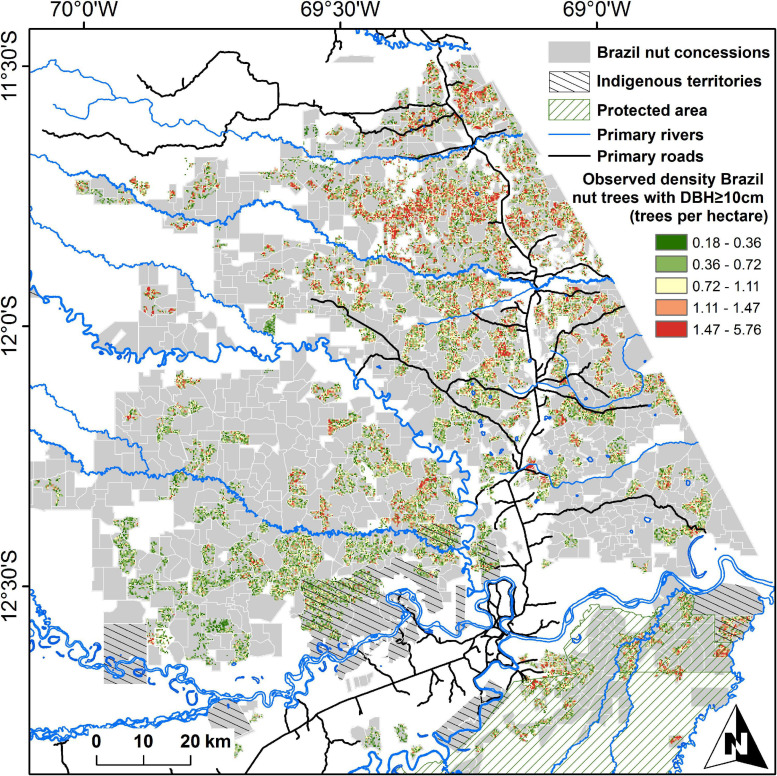
Distribution of Brazil nut concessions in Madre de Dios, Peru. Brazil nut tree densities [diameter at breast height (DBH) ≥ 10 cm] in concessions considered in this paper are presented as average numbers of trees per hectare, calculated from projections of single trees on 7.5-arc-second grid cells. Gray areas in sampled concessions had zero densities.

We used georeferenced data of 192,849 Brazil nut trees with diameter at breast height (DBH) ≥ 10 cm from 544 Brazil nut concessions in Madre de Dios, Peru, with an average size of 761 ha (range 164–575 ha; Figure 3). Data were collected from 2003 to 2007, in response to the Peruvian Forestry Law No. 27308 (5/10/2001), which obliged concession holders for the first time to present detailed inventories of the Brazil nut trees under their custody. The inventories were carried out by institutions active in the region, most notably ACCA (Asociación para la Conservación de la Cuenca Amazónica), CAMDE (Conservación Ambiental y Desarrollo en el Perú), FONDEBOSQUE (Fondo de Promoción del Desarrollo Forestal), AIDER (Asociación para la Investigación y el Desarrollo Integral), RNTAMB PRMRFFS (Programa Regional de Manejo de Recursos Forestales y Fauna Silvestre), Forestal Rio Piedras SAC, and Conservation International. Field staff from these institutions georeferenced individual trees, for the majority of these trees measured height and DBH (120,105 trees), and asked Brazil nut harvesters who accompanied them to estimate the average productivity of each individual tree (135,528 trees). For the vast majority of trees, these data were complemented with a description of each tree’s phytosanitary condition, by indicating whether a tree was covered by lianas, had broken branches or holes in its trunk, or showed evidence of termite nests, wound exudate, or tumors. Brazil nut seeds are harvested by cracking open the lignified capsular fruits with a machete after they have fallen on the ground. Individual seeds are protected by wooden testa, but these are not opened in the field, and harvesters express seed production weight in terms of “latas” (tin cans), which contain approximately 11.66 kg of fresh in-shell seeds. Previous analyses carried out with this dataset have demonstrated that seed production estimations by harvesters are reliable proxies of average production levels and that the data are robust enough to allow ecological hypothesis testing (Thomas et al., 2017, 2018). Annual seed production estimates of trees (SP_t_) varied from 0 to 362 kg, with an average of 30.3 ± 26.9 (SD) kg per tree. Approximately 13% of all trees were claimed never to produce by concessionaires. We used the DBH-based equations from Chave et al. (2014) to calculate AGB of individual Brazil nut trees (AGB_t_) using a wood density value of 0.59 g cm^–3^ (Chudnoff, 1984). AGB_t_ varied from 18 kg to 121.90 tons per tree, with an average value of 10.25 ± 8.07 tons. The relation between AGB_t_ and SP_t_ was unimodal to asymptotic (Thomas et al., 2017).

Low-intensity timber extraction, mostly focusing on selected timber species such as *Cedrela odorata* and *Swietenia macrophylla*, was prevalent in Brazil nut-rich forests several decades before the concession system was established and logging was formalized by a government decree in 2004 (Rockwell et al., 2017). However, the logging does not seem to have influenced Brazil nut regeneration patterns (Rockwell et al., 2017). This, together with the absence of high-density stands of adult Brazil nuts in the concession system (Thomas et al., 2015; Porcher et al., 2018), suggests that anthropogenic influences on Brazil nut tree density, seed production, and AGB have been minimal.

The environmental variables considered for explaining patterns in tree density, estimated AGB, and seed production were as follows: closeness to rivers and roads (binary variables; see below); all bioclimatic variables extracted from WorldClim climate layers (Hijmans et al., 2005); elevation; slope; terrain ruggedness index; and six major edaphic variables [organic carbon (ORCDRC), pH in H_2_O (PHIHOX), sand % (SNDPPT), silt % (SLTPPT), clay % (CLYPPT), and bulk density (BLD)] obtained from ISRIC-World Soil Information (Hengl et al., 2014). For the edaphic variables, we calculated a weighted mean across 0–5, 5–15, 15–30, 30–60, and 60–100 cm of soil depth values in order to derive a single data value for 0–100 cm. Table 1 gives an overview of the environmental gradients covered by the Brazil nut trees included in our dataset. It shows that while the study area is relatively small, the environmental gradients are wide enough for the purpose of this paper.

**TABLE 1:** Ranges of considered environmental variables in the study area.

	Min	Max
Bio1—Annual temperature (°C)	24.3	25.6
Bio2—Mean diurnal range (°C)	10.6	11.4
Bio3—Isothermality	66	75
Bio4—Temp seasonality	973	1,182
Bio5—Max temp warmest month (°C)	31.5	33.0
Bio6—Min temp coldest month (°C)	15.2	17.6
Bio7—Annual temp range (°C)	14.2	16.9
Bio8—Mean temp wettest quarter (°C)	25.0	26.4
Bio9—Mean temp driest quarter (°C)	22.6	24.0
Bio10—Mean temp warmest quarter (°C)	25.2	26.6
Bio11—Mean temp coldest quarter (°C)	22.6	24.0
Bio12—Annual precipitation (mm)	1,794	3,093
Bio13—Precipitation wettest month (mm)	246	460
Bio14—Precipitation driest month (mm)	28	104
Bio15—Precipitation seasonality	46	56
Bio16—Precipitation of wettest quarter (mm)	724	1,268
Bio17—Precipitation of driest quarter (mm)	122	344
Bio18—Precipitation of warmest quarter (mm)	462	1,150
Bio19—Precipitation of coldest quarter (mm)	122	344
Sand content (%)	30.7	54.7
Clay content (%)	27.7	40.6
Silt content (%)	14.0	31.1
Bulk density (kg/m^3^)	1,239.2	1,551.8
Organic carbon (g/kg)	4.05	31.95
pH	4.29	5.48
Elevation (m.a.s.l.)	176	408
Slope (°)	0	7.5
TRI	0.38	36.88

### Statistical Analyses

The accuracy and precision of area-based ecosystem service assessments are influenced by the spatial resolution of the analysis, and in the case of Brazil nut unit sizes above 5 ha have been recommended (Thomas et al., 2018). We therefore carried out area-based comparisons at a 7.5-arc-second resolution (∼5.4 ha at the equator). We generated a Brazil nut tree density map by projecting all trees on a 7.5-arc-second raster map, considering only grid cells with ≥90% spatial overlap with the polygons of inventoried concession, resulting in 56,818 grid cells with data and 32,528 grid cells with at least one tree (Figure 3). Cells intersected by roads or rivers were assigned a value of 1, and other cells a value of 0.

We used the method of Clark and Evans (1954) to generate maps with custom-made scripts in R (R Core Team, 2015) of the spatial aggregation of Brazil nut trees, whereby a value of 1 indicates random patterns, more than 1 more even spacing patterns, and less than 1 aggregated patterns (Clark and Evans, 1954). We corrected for edge effects by excluding trees that were located closer to a grid cell edge than to their closest neighbor using average nearest neighbor distance calculations per grid cell. Raster maps of estimated AGB and seed production (AGB_a_ and SP_a_) were obtained by summing the corresponding ABG and seed production scores of individual trees per grid cell.

To assess the importance of environmental variables in explaining the variability in the six different response variables (SP_t_, AGB_t_, tree density and spatial aggregation, and SP_a_, and AGB_a_), we developed random forest models by means of the *cforest* function in the *party* package for R (Strobl et al., 2007) and calculated importance values with the *varimp* function. Random forest models are not influenced by collinearity of variables, which is the reason why we included all climate and soil variables, in spite of strong correlations between some of them (Supplementary Figures 1–3). We included tree size (height and DBH), phytosanitary (presence of broken branches, tumors, vines, holes, exudate, and termites), and conspecific neighborhood variables (distance to nearest conspecific neighbor, spatial aggregation, and tree density) in random forest models of the tree-centric variables (height and DBH only in the seed production model) to assess their relative importance compared with the environmental variables.

We assessed the nature of the relationship between each of the six response variables and the environmental variables using generalized linear mixed models (GLMMs). We constructed GLMMs with Poisson distribution and log link functions for the density, and tree-centric and area-based seed production models, which were expressed as numbers of trees and multiples of tin cans, respectively. To account for overdispersion in these models, we corrected the standard errors using quasi GLMMs, where the variance is given by the product of the mean and the dispersion parameter. For spatial aggregation and tree-centric and area-based AGB, we constructed GLMMs with a Gaussian distribution. The presence of positive spatial autocorrelation in model residuals (assessed by means autocorrelograms) was effectively accounted for by including the concession where each tree was sampled as a random effect variable. GLMMs were implemented using Penalized Quasi-Likelihood in the MASS package for R (Venables and Ripley, 2002). Considering that different climate variables for different periods throughout the year (cf. Figure 2), in spite of being collinear, may generate different responses in tree-centric and area-based Brazil nut response variables, we retained all variables in the analysis.

## Results

**FIGURE 4:**
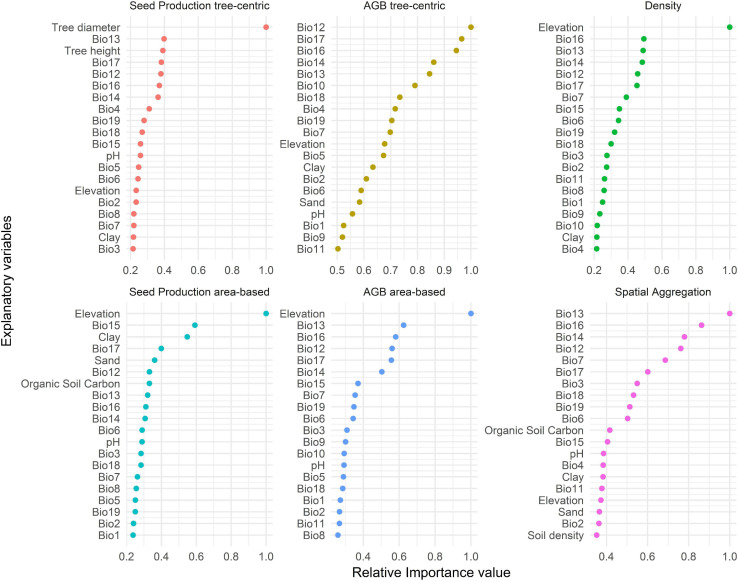
Relative importance values of the 15 most important variables for explaining variation in the six response variables. Bio1–11 correspond to temperature-related variables; Bio12–19 to precipitation-related variables. Bio1, annual mean temperature; Bio2, mean diurnal range; Bio3, isothermality (Bio2/Bio7) (100×); Bio4, temperature seasonality; Bio5, max temperature of warmest month; Bio6, min temperature of coldest month; Bio7, temperature annual range (Bio5–Bio6); Bio8, mean temperature of the wettest quarter; Bio9, mean temperature of the driest quarter; Bio10, mean temperature of the warmest quarter; Bio11, mean temperature of the coldest quarter; Bio12, annual precipitation; Bio13, precipitation of the wettest month; Bio14, precipitation of the driest month; Bio15, precipitation seasonality; Bio16, precipitation of the wettest quarter; Bio17, precipitation of the driest quarter; Bio18, precipitation of the warmest quarter; Bio19, precipitation of the coldest quarter.

Variable importance scores of random forest models revealed that precipitation variables (Bio12–19) were generally more important predictors of variation in all response variables than temperature (Bio1–11) and soil variables (Figure 4). Of the terrain variables, only elevation was among the 15 most important predictors of all response variables, and the most important for tree density and SP_a_ and AGB_a_. Of the tree-specific phytosanitary and size variables, only diameter and height were among the most important predictors of SP_t_. The suites of key predictors were relatively similar for all response variables, but their order varied considerably across response variables, albeit less so for tree density and AGB_a_.

Generalized linear mixed models characterizing the relationships between each of the six response variables and environmental gradients yielded low *R*^2^ values but allowed for the identification of significant signals of environmental filtering (Supplementary Figures 4–27). Responses to precipitation gradients generally had steeper slopes than the responses to most temperature variables, supporting the finding of the random forest modeling that precipitation variables were generally more important than temperature variables in explaining variation in the response variables. While the collinear temperature variables (Bio1 and 8–11) showed very similar patterns (in spite of differences in strength of correlations), responses to the collinear precipitation variables (Bio12–14 and 16–19) were much more variable (Table 2).

**TABLE 2:** Relations between environmental variables and Brazil nut tree fitness variables, densities and spatial aggregation, and tree- and area-based AGB and estimated seed production.

		Tree-centric habitat quality measures (fitness)		Area-based habitat quality measure		Area-based ecosystem services	
Corresponding time period		SP_t_	AGB_t_	Tree density	Spatial aggregation	SP_a_	AGB_a_
	• Annual temperature (Bio1)*	↓↓↓	↓↓↓	↓↓↓	↓↓↓	↓↓↓	↓↓↓
	• Mean diurnal range (Bio2)	↑↑↑	–	↑↑↑	↑↑↑	↑↑↑	↑↑↑D
	• Isothermality (Bio3)	↑↑↑	↑↑↑	↓↓↓	↓↓↓	– N	↓ D
	• Temp seasonality (Bio4)	↓↓↓	↓↓	↑↑↑	↑↑↑	– N	– N
September	• Max temp warmest month (Bio5)	↓↓↓	↓↓↓	↓↓↓	↑↑	↓↓↓	↓↓↓
July	• Min temp coldest month (Bio6)	↓↓↓	↑↑	↓↓↓	↓↓↓	↓↓↓	↓↓↓D
	• Annual temp range (Bio7)	–	↓↓↓	↑↑↑	↑↑↑	↑↑↑D	↑↑↑D
December–February	• Mean temp wettest quarter (Bio8)*	↓↓↓	↓↓↓	↓↓↓	↓↓	↓↓↓	↓↓↓
June–August	• Mean temp driest quarter (Bio9)*	↓↓↓	↓	↓↓↓	↓↓↓	↓↓↓	↓↓↓
September–November	• Mean temp warmest quarter (Bio10)*	↓↓↓	↓↓↓	↓↓↓	↓	↓↓↓	↓↓↓
May–July	• Mean temp coldest quarter (Bio11)*	↓↓↓	↓↓	↓↓↓	↓↓↓	↓↓↓	↓↓↓
	• Annual precipitation (Bio12)^#^	↑	↑↑↑	↓↓↓	↓↓↓	– N	↓↓↓D
January	• Precipitation wettest month (Bio13)^#^	↑↑	↑↑↑	↓↓↓	↓↓↓	– N	↓↓↓D
July	• Precipitation driest month (Bio14)^#^	↑↑↑	↑↑	↓↓↓	↓↓↓	↓D	↓↓↓D
	• Precipitation seasonality (Bio15)	↑↑↑	↓	↑↑↑	↑↑↑	↑↑↑	↑↑↑D
December–February	• Precipitation of wettest quarter (Bio16)^#^	↑↑	↑↑↑	↓↓↓	↓↓↓	– N	↓↓↓D
June–August	• Precipitation of driest quarter (Bio17)	↑↑↑	–↑	↓↓↓	↓↓↓	↓D	↓↓↓D
September–November	• Precipitation of warmest quarter (Bio18)	–	–	↓↓↓	↓↓↓	– F	–↓D
May–July	• Precipitation of coldest quarter (Bio19)^#^	↑↑↑	↑↑	↓↓↓	↓↓↓	– N	↓D
	• %Sand	↓↓↓	–	–↑	↑↑↑	↓↓F	– F
	• %Clay	↑↑	–	–	↓↓↓	↑F	–
	• %Silt	↑↑↑	–	–	↓↓	↑↑↑F	–
	• Bulk density	↑	–	↑↑↑	↑↑↑	–	– F
	• Organic carbon	↑↑	↑	↓↓↓	↓↓↓	– N	– N
	• pH	↓↓↓	↓↓↓	↑↑↑	↑↑↑	– N	– N
	• Elevation	↑↑↑	↑↑↑	↑↑↑	↑↑↑	↑↑↑	↑↑↑
	• Slope	↓↓↓	↓↓↓	↓↓↓	–	↓↓↓	↓↓↓
	• TRI	↓↓↓	↓↓↓	↓↓↓	–	↓↓↓	↓↓↓

–↑ and –↓ P ≤ 0.1; ↑ and ↓ P ≤ 0.05; ↑↑ and ↓↓ P ≤ 0.01; ↑↑↑ and ↓↓↓ P ≤ 0.001.

*Colinear temperature variables; ^#^colinear precipitation variables.

*D correlation parallels density trend; F correlation follows fitness (AGB_t_ or SP_t_) trend; N opposite density and tree-centric trends neutralize each other.*

*Phenological data are based on the authors’ long-term observations of Brazil nut phenology in the study region.*

*Area-based comparisons are based on 7.5-arc-second grid cells. Positive and negative correlations are indicated by “↑” and “↓,” respectively. Non-significant correlations are indicated by “–”. For ease of interpretation, correlations for spatial aggregation are the inverse of those obtained based on Clark and Evan’s method (randomness).*

**FIGURE 5:**
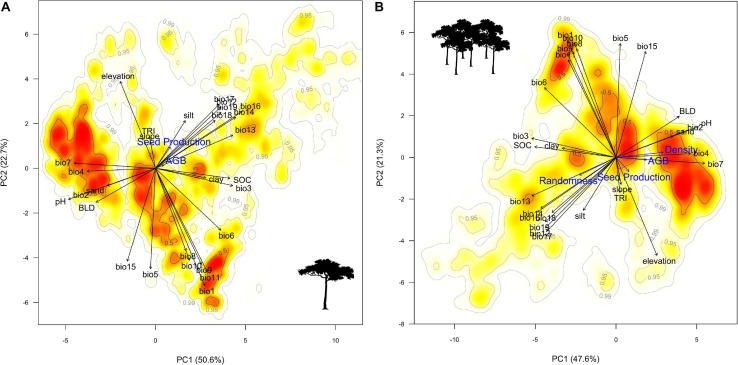
Ordination plots showing the first two axes of principal component analyses of tree-centric Brazil nut variables **(A)** and area-based Brazil nut variables **(B)**. The color gradient indicates regions of the highest (red) to lowest (white) densities of data points, i.e., individual Brazil nut trees in **(A)** and 7.5-arc-second grid cells in **(B)**, through use of Kernell density estimations. Please note that randomness is inversely related with spatial aggregation. Arrows of the response variables were doubled **(B)** to tripled **(A)** in length to improve interpretation. Bio1, annual mean temperature; Bio2, mean diurnal range; Bio3, isothermality (Bio2/Bio7) (100×); Bio4, temperature seasonality; Bio5, max temperature of the warmest month; Bio6, min temperature of the coldest month; Bio7, temperature annual range (Bio5–Bio6); Bio8, mean temperature of the wettest quarter; Bio9, mean temperature of the driest quarter; Bio10, mean temperature of the warmest quarter; Bio11, mean temperature of the coldest quarter; Bio12, annual precipitation; Bio13, precipitation of the wettest month; Bio14, precipitation of the driest month; Bio15, precipitation seasonality; Bio16, precipitation of the wettest quarter; Bio17, precipitation of the driest quarter; Bio18, precipitation of the warmest quarter; Bio19, precipitation of the coldest quarter.

**FIGURE 6:**
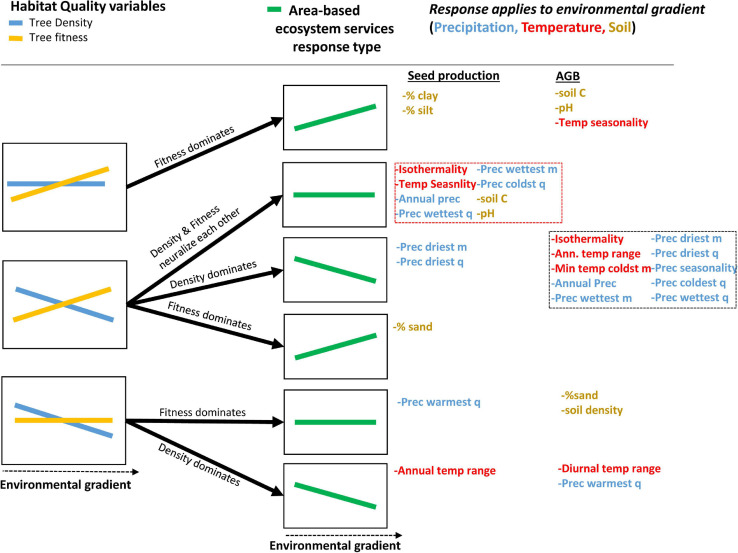
Relations between area-based ecosystem services (SP_a_ and AGB_a_) and environmental gradients (green line graphs), as the net result of relations between tree-centric (SP_t_ and AGB_t_) and area-based (tree density) habitat quality variables (orange and blue line graphs) and those environmental gradients. The black arrows indicate which of the habitat quality variables (i.e., fitness or density) dominated the relation between area-based ecosystem services and the environmental gradients corresponding to the different climate, soil, and terrain variables listed on the right-hand side. Only environmental gradients that generated non-congruent responses of tree-centric and area-based habitat quality variables are represented (see Table 2). Note that “flat” lines indicate non-significant relations, while “inclined” lines indicate significant relations, but these can refer to either a positive or negative relation. For meaning of dashed red and black boxes, please refer to the text. As an example, the response to annual precipitation (listed in dashed red box on the right-hand side) of area-based seed production is invariable (2nd green graph from top), which is the result of the positive and negative responses of density and tree-centric seed production, respectively, which neutralize each other (central graph on the left-hand side). By contrast, annual precipitation (listed in lower dashed black box on the right-hand side) generates a negative response of area-based AGB (3rd green graph from top), because the negative correlation of tree density with precipitation dominates the positive one of tree-centric AGB (central graph on the left-hand side). This same example is also illustrated in Figure 7.

Tree-centric habitat quality measures (SP_t_ and AGB_t_) showed congruent responses to most (but not all) of the environmental variables tested but yielded opposite trends to Brazil nut tree density in about half of the cases, even so when excluding collinear variables with similar responses (Table 2, Figure 5, and Supplementary Figures 4–27). As expected, density and spatial aggregation of Brazil nut trees yielded almost identical responses (Figure 5), suggesting that both are controlled by similar ecological processes. Opposite responses of tree-centric and area-based habitat quality measures to environmental variables (middle left graph Figure 6) resulted in predominantly neutralizing effects for relationships between environmental variables and SP_a_ (non-significant relations in eight out of 11 cases; dashed red box Figure 6). By contrast, responses of AGB_a_ strongly mirrored those of tree density (10 out of 13 cases; dashed black box Figure 6).

## Discussion

**FIGURE 7:**
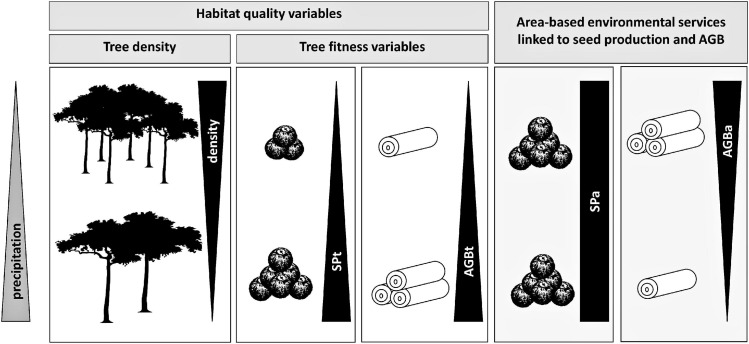
Graphical illustration of how different habitat quality variables and area-based ecosystem services, linked to seed production and AGB of Brazil nut, respond to a precipitation gradient. The positive and negative correlations with precipitation of SP_t_ and tree density, respectively, neutralize one another, resulting in invariable SP_a_ regardless of precipitation levels. By contrast, the negative correlation of tree density with precipitation dominates the positive one of AGB_t_, resulting in a negative correlation of AGB_a_.

Our objective was to assess the existence of habitat quality differentiation between individuals and stands of Brazil nut trees and to identify the environmental drivers contributing to this differentiation. Our findings show that the environmental gradients we considered generated approximately 50% congruent and 50% divergent responses of fitness variables [seed production (SP_t_) or aboveground biomass (AGB_t_)] on the one hand and tree density on the other. In the case of divergent responses, we expected environmental filters on SP_a_ and AGB_a_ to mirror those of tree density, but this was only the case for AGB_a_. In contrast, relations between environmental variables and SP_a_ tended to be predominantly neutralized by the opposite responses of SP_t_ and tree density (Figure 7). Hence, while we accept our first hypothesis (that the *responses of area-based ecosystem service production by the Brazil nut to environmental gradients mirror those of its density*) for AGB, we partly reject it for seed production. The trade-offs created by multiple climate and soil gradients between density and fitness of Brazil nut trees on the other hand are in line with our second hypothesis (*density and aggregation responses to gradients are governed by expectations under the Janzen–Connell and Herms–Mattson hypotheses, whereas fitness responses are in line with niche preferences and resource requirements of trees across their life cycles*). In the following sections, we discuss how our findings are in support of these hypotheses. We conclude with a discussion of implications of our findings and suggest avenues of future research.

### Climate-Driven Habitat Quality Differentiation

Our findings suggest that higher precipitation volumes in different periods of the year positively influence Brazil nut fitness variables but tend to decrease tree density and aggregation. This is in line with the Janzen–Connell hypothesis, which predicts Brazil nut tree recruitment to be lower and less aggregated in more rainy areas due to stronger CNDDD effects associated with increased pest and disease pressure on seedlings and saplings under wetter conditions (Swinfield et al., 2012; Comita et al., 2014; Uriarte et al., 2018). Pest- and disease-related mortality in Brazil nut in the study region is likely to occur during the transition from small (<50 cm) to large seedlings (50–150 cm) due to aerial pest and diseases, soil-borne pathogens, or both (Porcher et al., 2018).

While lower precipitation levels were associated with higher Brazil nut density, AGB_t_ and SP_t_ tended to be lower. Water limitation is increasingly found to constrain tree growth in forests around the world and disproportionately so in the largest trees (Bennett et al., 2015; Babst et al., 2019). Adult Brazil nut trees are canopy emergents and hence likely to be more vulnerable to hydraulic stress and experience higher radiation and evaporative demand because of exposed crowns (Bennett et al., 2015). Rainfall was among the most important variables positively influencing basal area increment and seed production of Brazil nut trees in Acre, Brazil (Kainer et al., 2007; Staudhammer et al., 2013). However, while precipitation during the dry season was strongly related to SP_t_ (Supplementary Figure 5 and Kainer et al., 2007), we found it to be only weakly correlated with AGB_t_. We suggest that this may be due to this being the period Brazil nut trees shed leaves and hence photosynthetic activity and stem growth slows down, whereas fruit growth is at its midpoint (Figure 2) (Cavalcante, 1991; Schöngart et al., 2015).

Higher air temperatures in different periods of the year tended to negatively influence both tree-centric and area-based Brazil nut variables, which again is in line with expectations. The Janzen–Connell hypothesis predicts that enemy-mediated conspecific effects are amplified under warmer conditions and hence lead to lower conspecific tree density and aggregation (Swinfield et al., 2012; Comita et al., 2014). In line with our findings, Thompson et al. (2010) found that fungus-induced mortality of seedlings of the Amazonian hyperdominant palm *Iriartea deltoidea* increased with air temperature. On the other hand, the positive effect of temperature on pathogen proliferation only plays within certain temperature ranges, while extreme temperatures have been shown to inhibit growth and spore germination of fungal pathogens (Copes and Hendrix, 2004; Thompson et al., 2010), particularly in combination with low humidity (Talley et al., 2002). This might explain why we found a negative correlation between the spatial aggregation of Brazil nut trees and the maximum temperature of the warmest month, which follows the driest quarter of the year in the study region.

Our results further add support to the growing body of evidence on the negative effects of higher temperatures on the fitness of tropical tree species (Feeley et al., 2007; Way and Oren, 2010; Wagner et al., 2014). Elevated day temperatures may reduce photosynthetic rates in trees due to vapor pressure deficits (the relative dryness of the air), while higher minimum (nighttime) temperatures may inflate respiratory costs (Clark et al., 2013; Allen et al., 2015; Fontes et al., 2018). Negative responses of SP_t_ to higher temperatures, particularly during the warmest and wettest quarter, may be because these periods coincide with the periods of flowering and fruit set of Brazil nut (Figure 2) (Corvera-Gomringer et al., 2010). Exposure to extreme temperatures during the pollination stage, or initial seed or fruit set, is generally expected to reduce yield potential (Redmond et al., 2012; Hatfield and Prueger, 2015). High temperatures have also been found to increase flower and fruit abortion, particularly during droughts (Leite et al., 2007; Pérez-Ramos et al., 2010; Girard et al., 2012; Hatfield and Prueger, 2015). There is anecdotal evidence from our study region, Madre de Dios, that a combination of high temperatures and droughts in 2005 and 2010 led to massive Brazil nut flower abortion (Thomas et al., 2017).

Higher variability in precipitation and temperature regimes on daily and annual bases generated more variable responses in tree-centric and area-based Brazil nut variables. In line with the Janzen–Connell hypothesis that pest- and disease-related mortality of conspecifics is favored not only by wetter and hotter but also by more stable climate conditions, this explains why the highest Brazil nut tree densities and aggregations tended to be located in areas with higher precipitation and temperature seasonality, as well as higher diurnal and annual temperature ranges. The response of fitness variables on the other hand tended to be governed by niche preferences and resource requirements of Brazil nut trees across their life cycles. AGB_t_ was positively influenced by more stable and moderate precipitation and temperature regimes, i.e., lower variability and extremes on seasonal and yearly bases. Higher precipitation seasonality in the tropics has been found to limit leaf carbon assimilation and wood production of trees (Saatchi et al., 2007; Wagner et al., 2016), which in the case of Brazil nut might translate in lower AGB_t_. Both colder temperatures during the coldest month of the year and higher temperatures during the warmest periods of the year were associated with lower AGB_t_. The positive correlation between AGB_t_ and the minimum temperature of the coldest month was unexpected but could be related to acclimatization of nighttime respiration in response to mean growth temperature (Cheesman and Winter, 2013; Slot et al., 2014). Consistent with this, in a pan-tropical meta-analysis, Wagner et al. (2014) found minimal temperatures to be positively, albeit weakly, correlated with tree growth.

SP_t_ showed a more complex relation with temporal climate variability. Similar to AGB_t_, it responded positively to lower temperature seasonality and lower maximum temperatures during the warmest periods of the year but negatively to lower precipitation seasonality, which is in line with Müller’s (1981) argument that the Brazil nut requires 2–5 months with reduced rainfall for good fruit production. On the other hand, our finding that SP_t_ tended to be higher in areas with lower minimum temperatures of the coldest month and higher mean daily temperature ranges suggests that lower night temperatures favor fecundity, possibly in a similar manner as chilling requirements for flowering in temperate fruit trees (Guo et al., 2014).

### Soil-Driven Habitat Quality Differentiation

Our findings further suggest that also soil variables can generate habitat quality differentiation. Soils with lower fertility or less favorable rooting conditions tended to be associated with higher Brazil nut tree density and aggregation, in line with the Herms–Mattson hypothesis, but lower fitness variables, in line with the species’ niche preferences and resources requirements. According to the Herms–Mattson hypothesis, infertile soils or unfavorable rooting conditions tend to favor greater allocation to anti-herbivore defenses (secondary metabolites) in trees, which leads to lower rates of pest- and disease-related mortality, hence favoring higher conspecific densities (Givnish, 1999). This hypothesis was experimentally validated by Fine et al. (2006) in the Peruvian Amazon. Accordingly, we found Brazil nut density and aggregation to correlate negatively with soil organic carbon content, which is an indicator of soil fertility, supporting experimental findings from temperate forest that the strength of CNDDD recruitment of seedlings and saplings increased with soil resource availability (LaManna et al., 2016). Similarly, the positive correlation between soil pH and Brazil nut density and aggregation might be the consequence of the inverse correlation between pH and organic soil carbon in the study region (Supplementary Figure 3). However, at low pH, elevated levels of certain elements such as aluminum and manganese can reach toxic levels, which may be more difficult to cope with for seedlings and saplings than for adult trees (Delhaize and Ryan, 1995; Melakeberhan et al., 1995). Soils in the study area are known to have an elevated aluminum content (Quesada et al., 2011). In line with the Herms–Mattson hypothesis (Givnish, 1999), Brazil nut tree density and aggregation also correlated positively with the bulk density of the fine earth fraction, which is an indicator of how well plant roots are able to extend into the soil. The highest densities in our dataset occurred in areas with values >1.35 g/cm^3^ above which inhibition of root growth may start (Quesada et al., 2012).

By contrast, more fertile soil conditions tended to increase SP_t_ and AGB_t_, corroborating field research in Acre, Brazil. Staudhammer et al. (2013) documented a positive relation between soil carbon and basal area increment of Brazil nut trees, while CEC, being collinear with soil carbon, was found to positively influence fruit production of Brazil nut trees in the same study area (Kainer et al., 2007). The strong negative correlations we found between soil pH and SP_t_ and AGB_t_ might therefore be a reflection of the strong inverse correlation between pH and soil organic carbon in the study area (Supplementary Figure 3), although other factors may be important too. For example, more acidic soils in the Amazon have been found to contain higher soil calcium ion concentrations (Laurance et al., 1999), which in turn correlated positively with diameter increment of adult Brazil nut trees in Acre (Staudhammer et al., 2013). Similarly, the positive influence on SP_t_ by the clay and silt fraction of soils and the negative influence of the sand fraction might be an expression of the positive correlation between clay and silt content and soil organic carbon in the research area (Supplementary Figure 3).

Topographical variables such as elevation, slope, or landscape heterogeneity may influence variation in soil chemistry, hydrology, and microclimate, thus driving niche partitioning and environmental filtering of tree species in tropical forests (Douda et al., 2012; Brown et al., 2013; Jucker et al., 2018; Zuleta et al., 2018; Mutuku and Kenfack, 2019). However, we did not find evidence for topography causing habitat quality differentiation in the Brazil nut. Brazil nut prefers well-drained *terra firme* soils (Mori and Prance, 1990), which explains why SP_t_ and AGB_t_ increased with elevation. Negative correlations with terrain slope and heterogeneity (TRI) might on the other hand be related to decreasing availability of water and nutrients (Paoli et al., 2008), or the lower likelihood of large-sized Brazil nut trees to establish on hilly terrains. Topographic heterogeneity has been found to positively correlate with tree species richness (Kubota et al., 2004; Douda et al., 2012; Rodrigues et al., 2019). In less topographically heterogeneous forests, one would therefore expect higher abundances of at least some species, which could explain why we found higher Brazil nut tree densities on flatter terrain. Accordingly, Rodrigues et al. (2019) found not only lower diversity in less topographically heterogeneous Brazilian Atlantic forest but also a higher abundance of hyperdominant tree species.

### Avenues for Future Research

While further research is needed to test the validity of the mechanisms underlying the trade-offs between tree-centric and area-based habitat quality variables and quantify the variation in their strength across environmental gradients, previous work (Thomas et al., 2018) has shown that SP_t_ and AGB_t_ are conditioned by CNDDD effects. Environmental controls on tree density are therefore likely to indirectly influence measures of individual tree fitness. An interesting area for future research may be unraveling how the variable strength of CNDDD across environmental gradients leads to divergent responses of tree density and fitness variables.

Environmental variables that generate congruent responses of SP_t_ or AGB_t_ on the one hand and density on the other can be expected to weaken CNDDD of SP_t_ and AGB_t_, whereas environmental conditions that foster opposite responses are either expected to promote the strength of CNDDD or have no effect. For example, precipitation correlated positively with SP_t_ and AGB_t_ and negatively with tree density (Figure 7). At the same time, both tree-centric variables also correlated negatively with tree density (Thomas et al., 2018). Hence, in areas with lower precipitation, SP_t_ and AGB_t_ on average tended to be at the lower end while tree density tended to be at the higher end of the observed spectrum, which might further lower SP_t_ and AGB_t_ through CNDDD. In this example, precipitation would be considered an environmental variable strengthening CNDDD effects on SP_t_ and AGB_t_, analogous with comparable findings for CNDDD effects on recruitment (Comita et al., 2014; LaManna et al., 2016). Further research at a finer spatial scale is needed to test the validity of this hypothesis.

## Conclusion

We found that environmental filters generate trade-offs between tree-centric (fitness) and area-based (density) habitat quality indicators of Brazil nut, which are translated into divergent responses to multiple environmental gradients of area-based ecosystem services related to seed production and AGB. The fact that even within our relatively small study region environmental gradients were pronounced enough to cause these trade-offs suggests that even stronger trends might be detected across the entire species range.

Our finding that higher temperatures tended to affect all habitat quality variables of the Brazil nut negatively, while the influence of precipitation was more variable, but also more important, suggests that climate change is expected to impact the provision of ecosystem services. It is clear that multivariate modeling approaches are needed to obtain a comprehensive understanding of the nature of expected changes in ecosystem service provision by this giant of the Amazon and associated impacts on local livelihoods under climate change.

## Data Availability Statement

Publicly available datasets generated for this study. This data can be found here: https://doi.org/10.1371/journal.pone.0183743.s001.

## Author Contributions

ET designed the study, performed data curation and statistical analyses, and prepared the first draft of the manuscript. MJ, FC-A, LW, RC-G, RA, SB, MV-R, and BL contributed to revisions of the manuscript. All authors contributed to the article and approved the submitted version.

## Conflict of Interest

The authors declare that the research was conducted in the absence of any commercial or financial relationships that could be construed as a potential conflict of interest.
