# Supplementary material for: Habitat Quality Differentiation and Consequences for Ecosystem Service Provision of an Amazonian Hyperdominant Tree Species
Source: Front Plant Sci. 2021 Mar 31;12:621064. doi: 10.3389/fpls.2021.621064 (PMC8044455; doi:10.3389/fpls.2021.621064)
Supplement: Supplementary file 1 [file Data_Sheet_1.docx]

Supplementary Material

Habitat quality differentiation and consequences for ecosystem service provision of an Amazonian hyperdominant tree species

**Evert Thomas^1^*, Merel Jansen^2,3^, Fidel Chiriboga-Arroyo^2^, Lúcia H.O. Wadt^4^, Ronald Corvera-Gomringer^5^, Rachel Judith Atkinson^1^, Stephen P Bonser^6^, Manuel Gabriel Velasquez-Ramirez^5^, Brenton Ladd^7^**

^1^ Bioversity International, Lima, Peru, [evert.thomas@gmail.com](mailto:evert.thomas@gmail.com), r.atkinson@cgiar.org

^2^ ETH Zürich, Ecosystem Management group, Switzerland, [mereljansen2@gmail.com](mailto:mereljansen2@gmail.com), [fidel.chiriboga.a@usys.ethz.ch](mailto:fidel.chiriboga.a@usys.ethz.ch)

^3^ Center for International Forestry Research -CIFOR, Lima, Peru

^4^ Embrapa Rondônia, Porto Velho-RO, Brasil, [lucia.wadt@embrapa.br](mailto:lucia.wadt@embrapa.br)

^5^ Instituto de Investigaciones de la Amazonia Peruana – IIAP, Puerto Maldonado, Perú, [rcorvera@iiap.gob.pe](mailto:rcorvera@iiap.gob.pe), mvelasquez@iiap.gob.pe

^6^ School of Biological, Earth and Environmental Science & Ecology and Evolution Research Centre, UNSW Sydney, Australia, [s.bonser@unsw.edu.au](mailto:s.bonser@unsw.edu.au)

^7^ Escuela de Agroforestería, Universidad Científica del Sur; Lima 33, Perú, [bladd@cientifica.edu.pe](mailto:bladd@cientifica.edu.pe)

* **Correspondence**:

Evert Thomas

[evert.thomas@gmail.com](mailto:evert.thomas@gmail.com)

Brenton Ladd

[bladd@cientifica.edu.pe](mailto:bladd@cientifica.edu.pe)


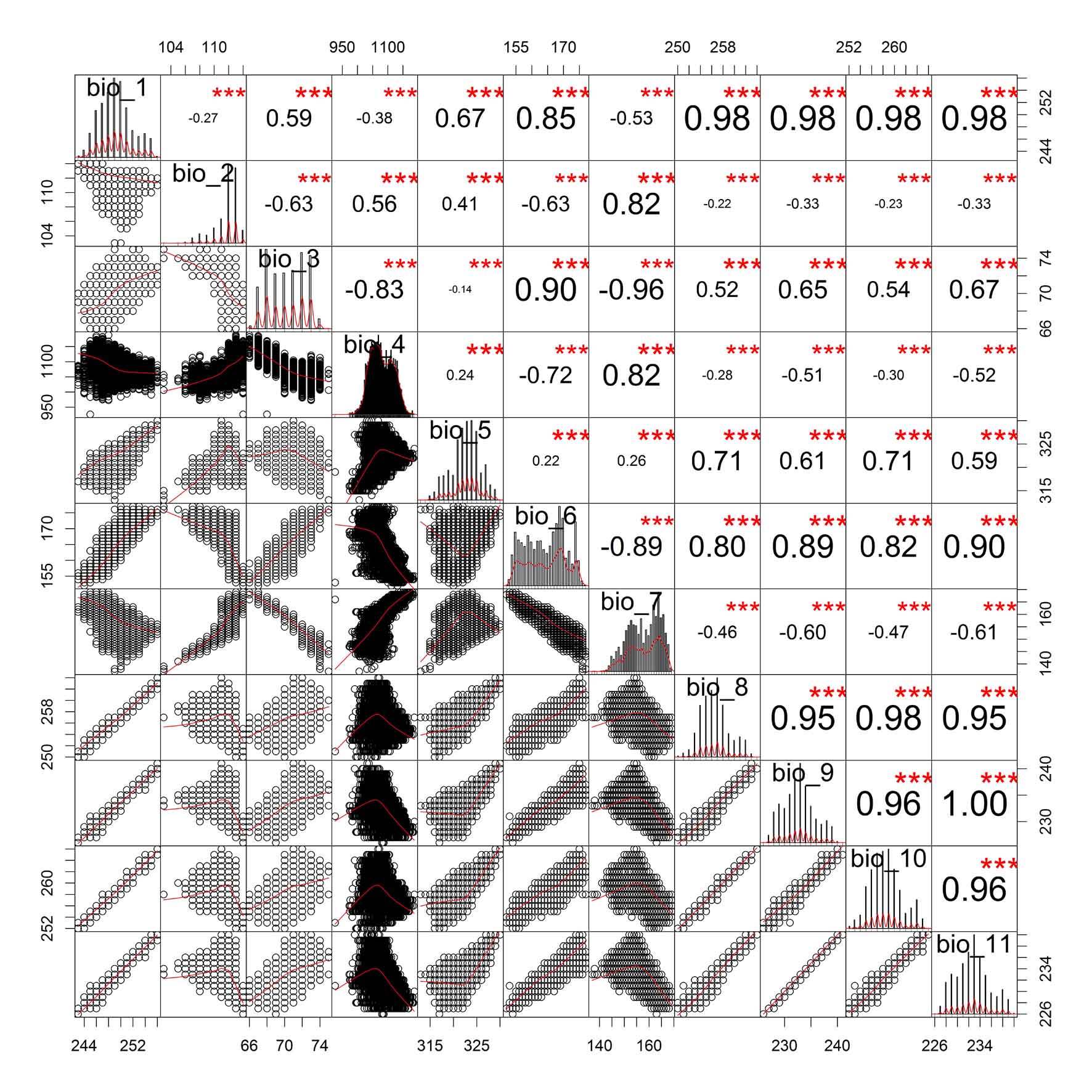


**Figure S1** Correlations between temperature variables


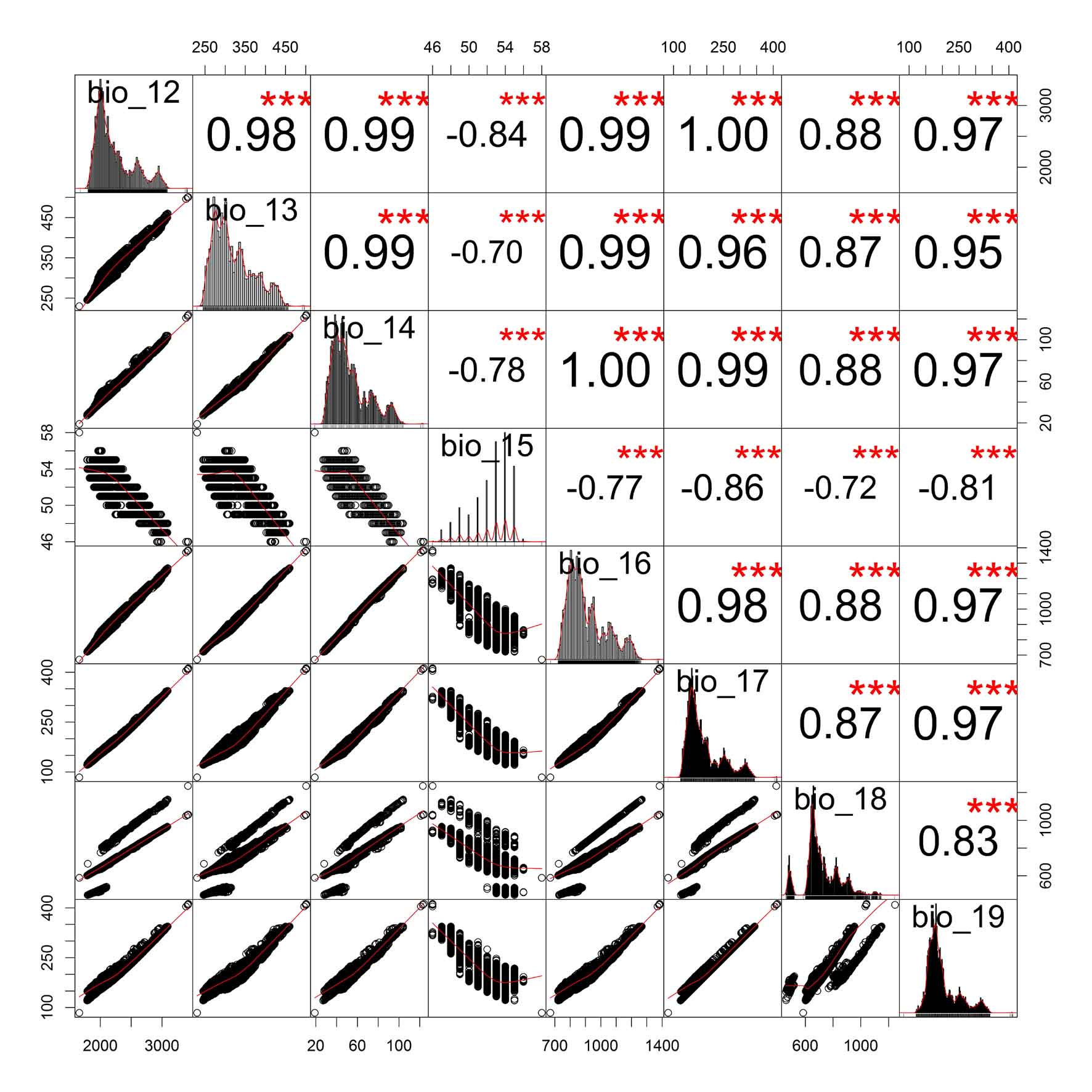


**Figure S2** Correlations between precipitation variables


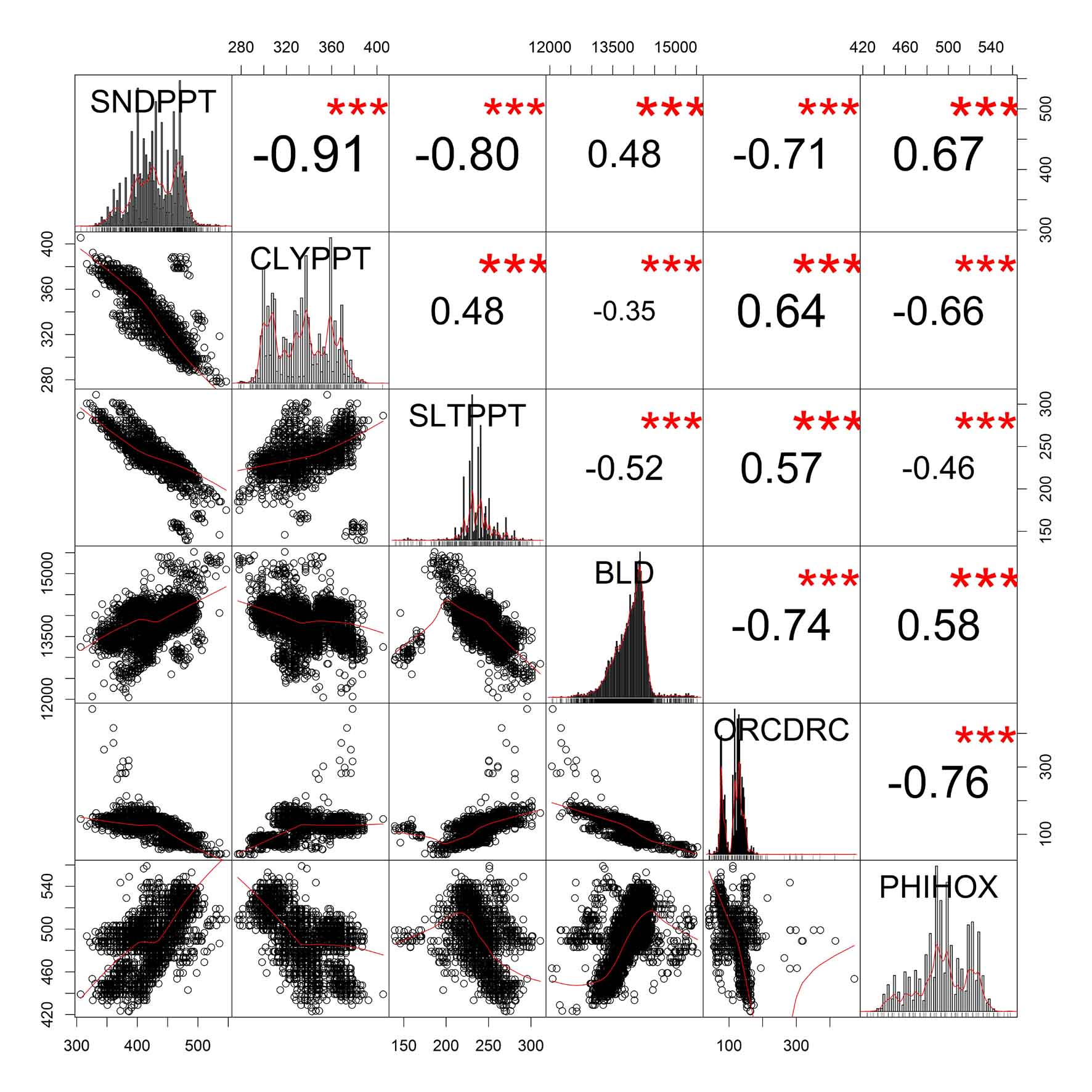


**Figure S3** Correlations between soil variables


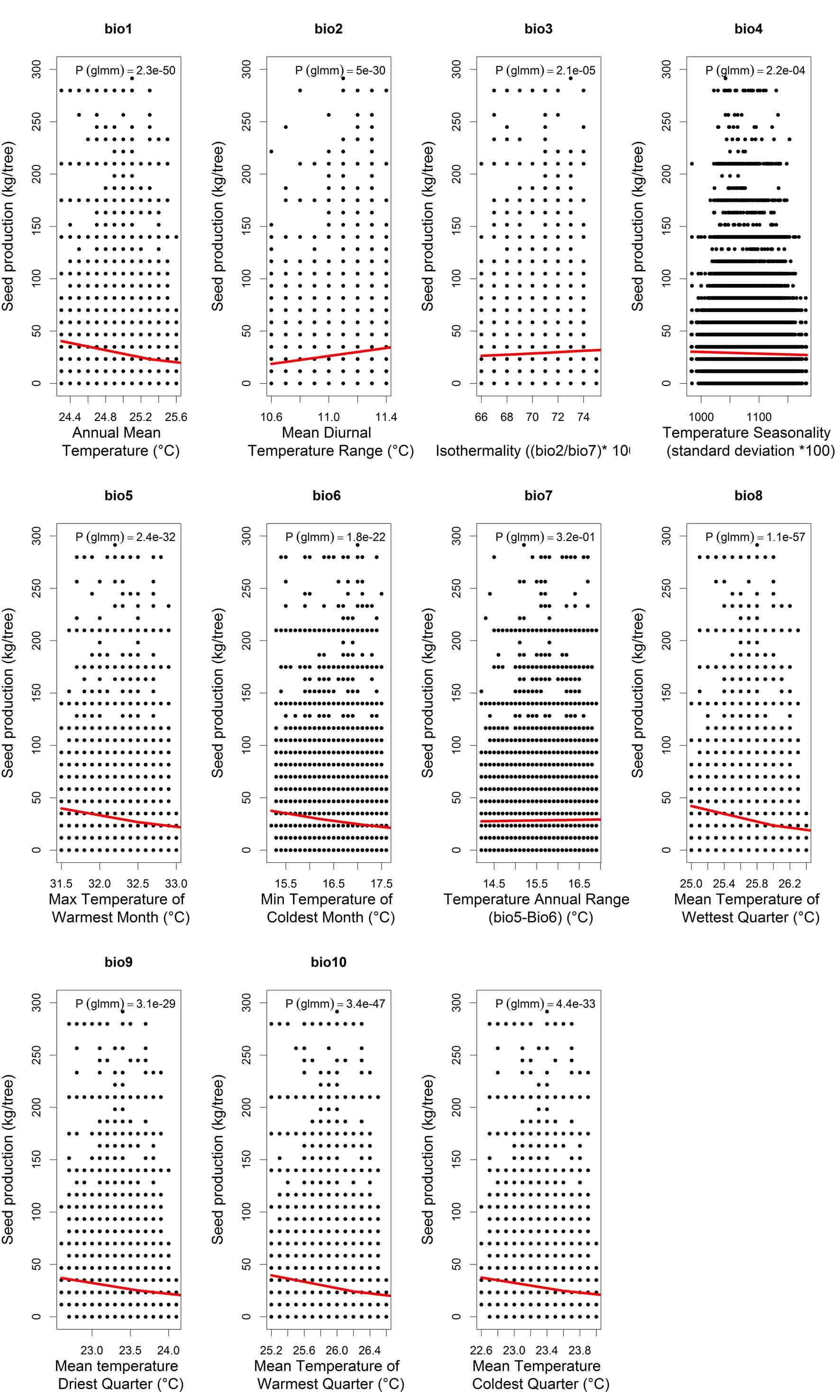


**Figure S4** Relation between estimated seed production of individual Brazil nut trees and air temperature variables. Please note that the average estimated seed production of all trees in our dataset is 30.3±26.9 (SD) kg per tree, and that seed production estimates are expressed as multiples of tin cans, corresponding with approximately 11.66kg.


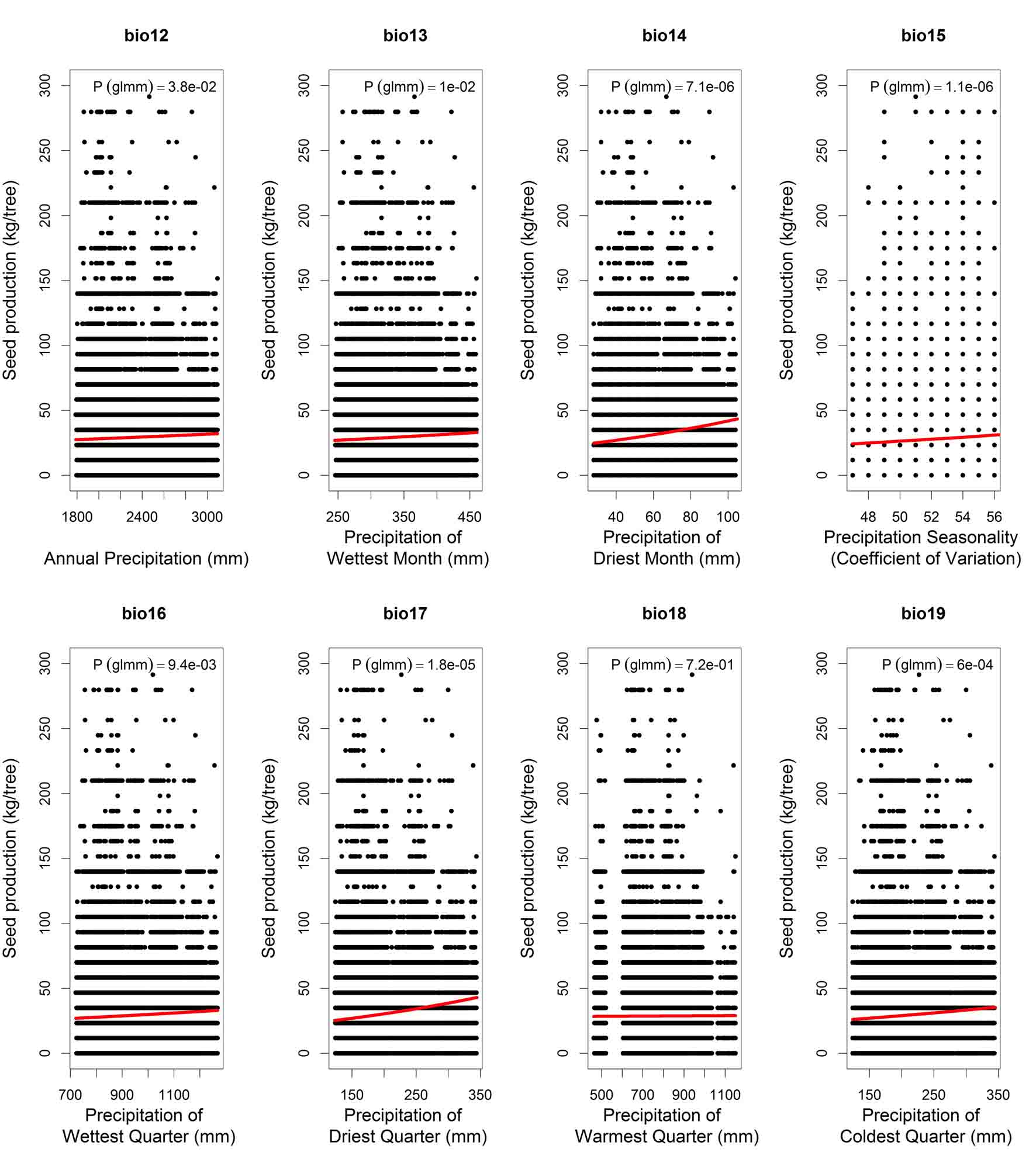


**Figure S5** Relation between estimated seed production of individual Brazil nut trees and precipitation variables. Please note that the average estimated seed production of all trees in our dataset is 30.3±26.9 (SD) kg per tree, and that seed production estimates are expressed as multiples of tin cans, corresponding with approximately 11.66kg.


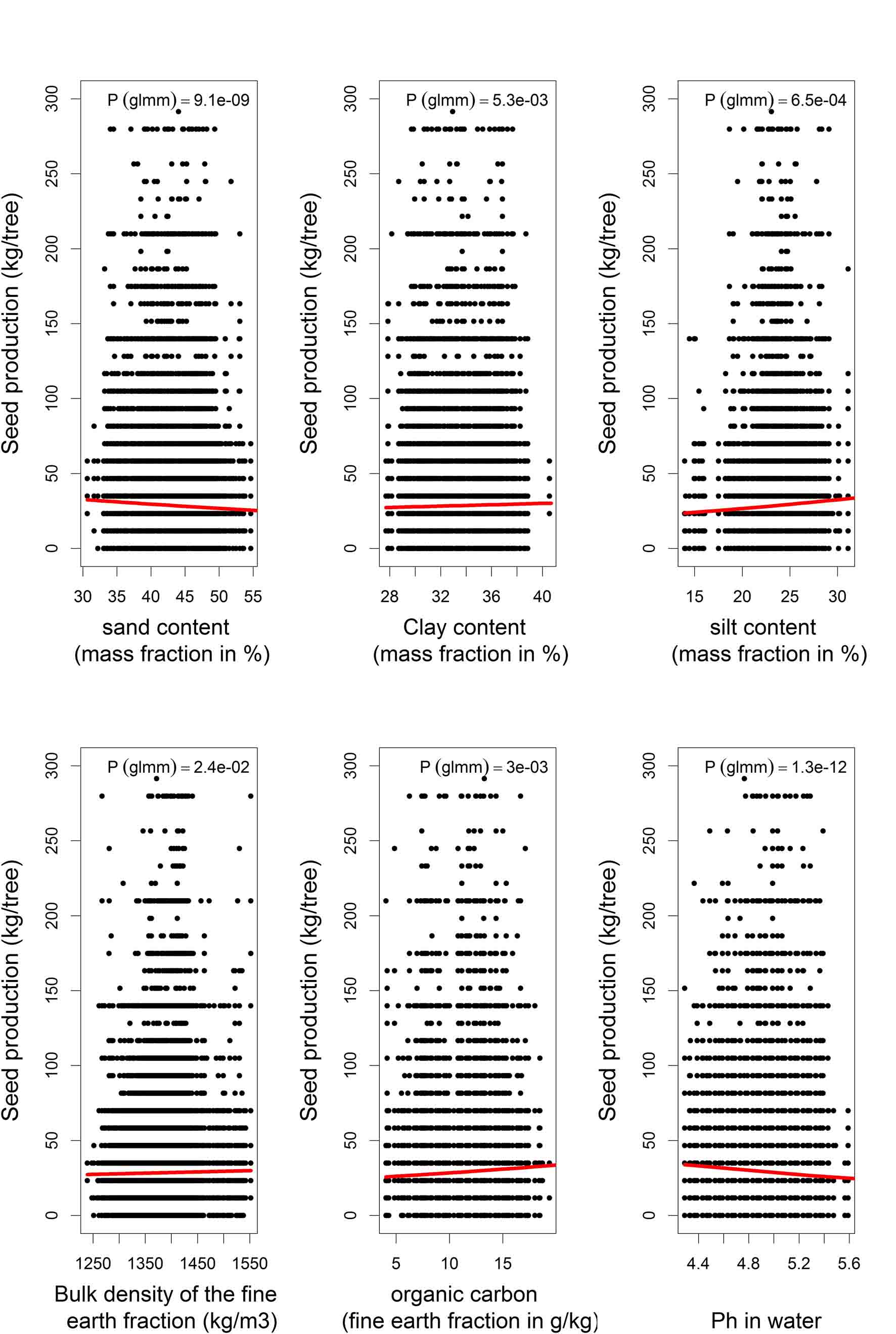


**Figure S6** Relation between estimated seed production of individual Brazil nut trees and soil variables. Please note that the average estimated seed production of all trees in our dataset is 30.3±26.9 (SD) kg per tree, and that seed production estimates are expressed as multiples of tin cans, corresponding with approximately 11.66kg.


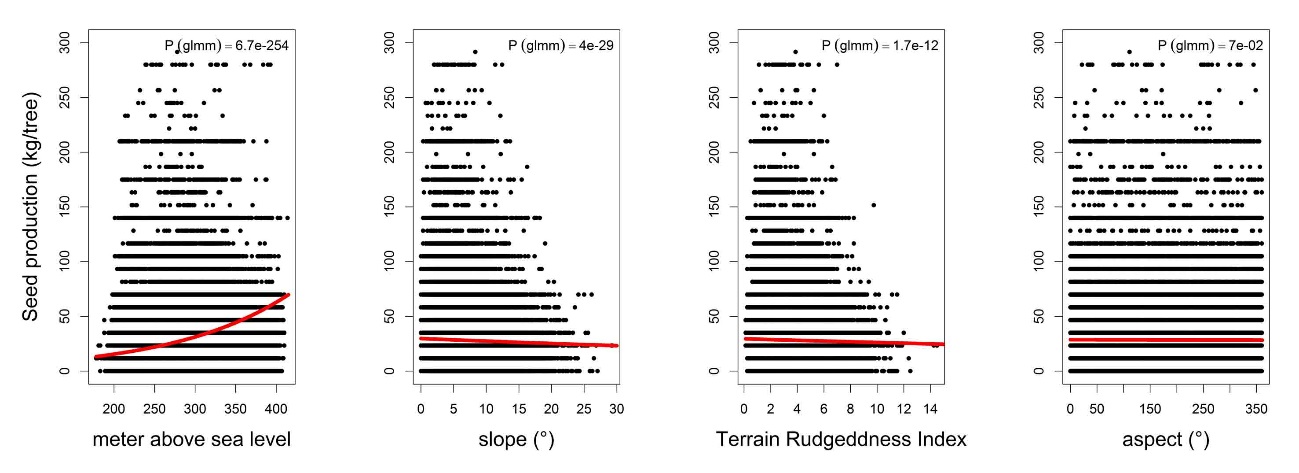


**Figure S7** Relation between estimated seed production of individual Brazil nut trees and terrain variables. Please note that the average estimated seed production of all trees in our dataset is 30.3±26.9 (SD) kg per tree, and that seed production estimates are expressed as multiples of tin cans, corresponding with approximately 11.66kg.


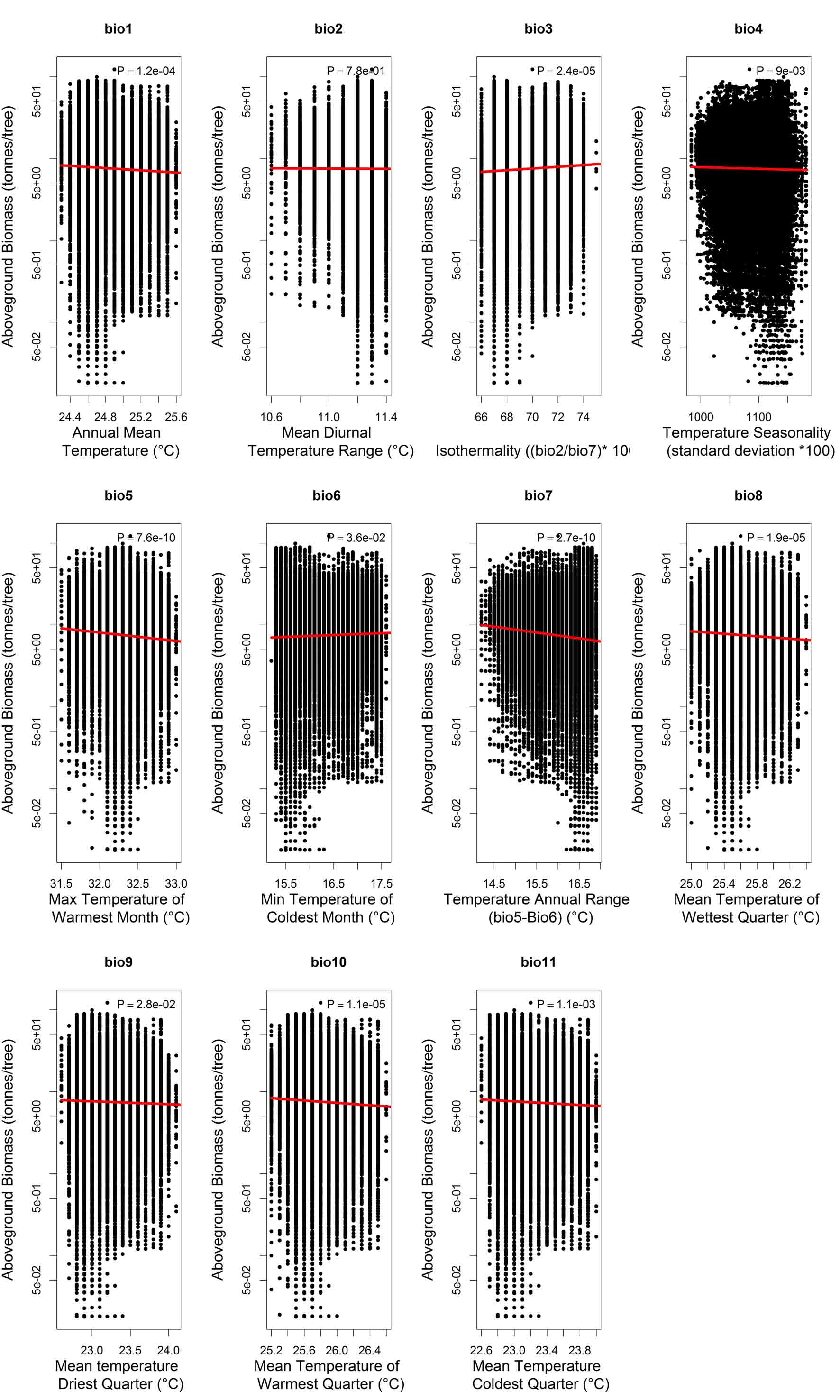


**Figure S8** Relation between estimated AGB of individual Brazil nut trees and air temperature variables. Please note that the average estimated AGB of all trees in our dataset was 10.25±8.07 tonnes per tree.


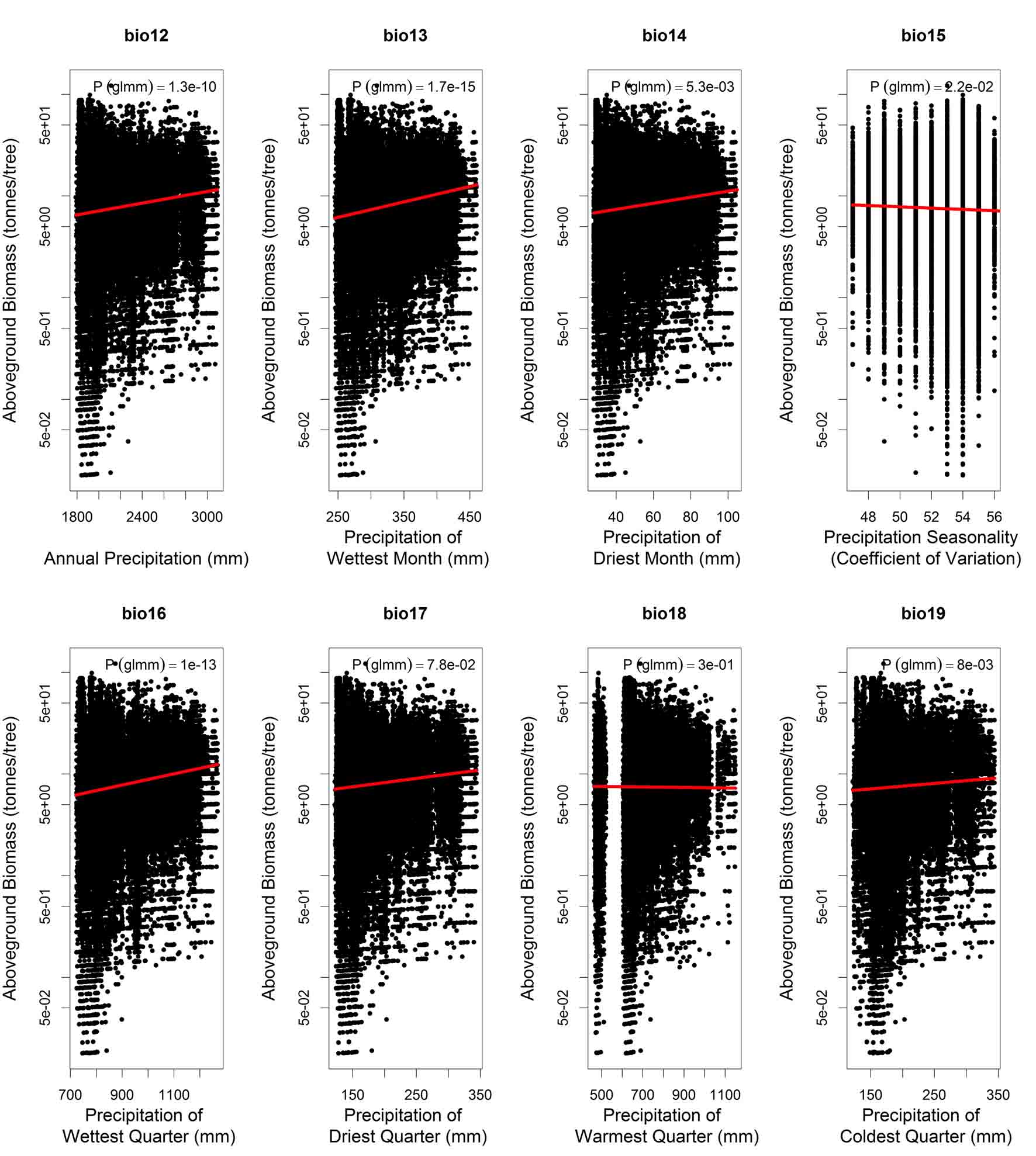


**Figure S9** Relation between estimated AGB of individual Brazil nut trees and precipitation variables. Please note that the average estimated AGB of all trees in our dataset was 10.25±8.07 tonnes per tree.


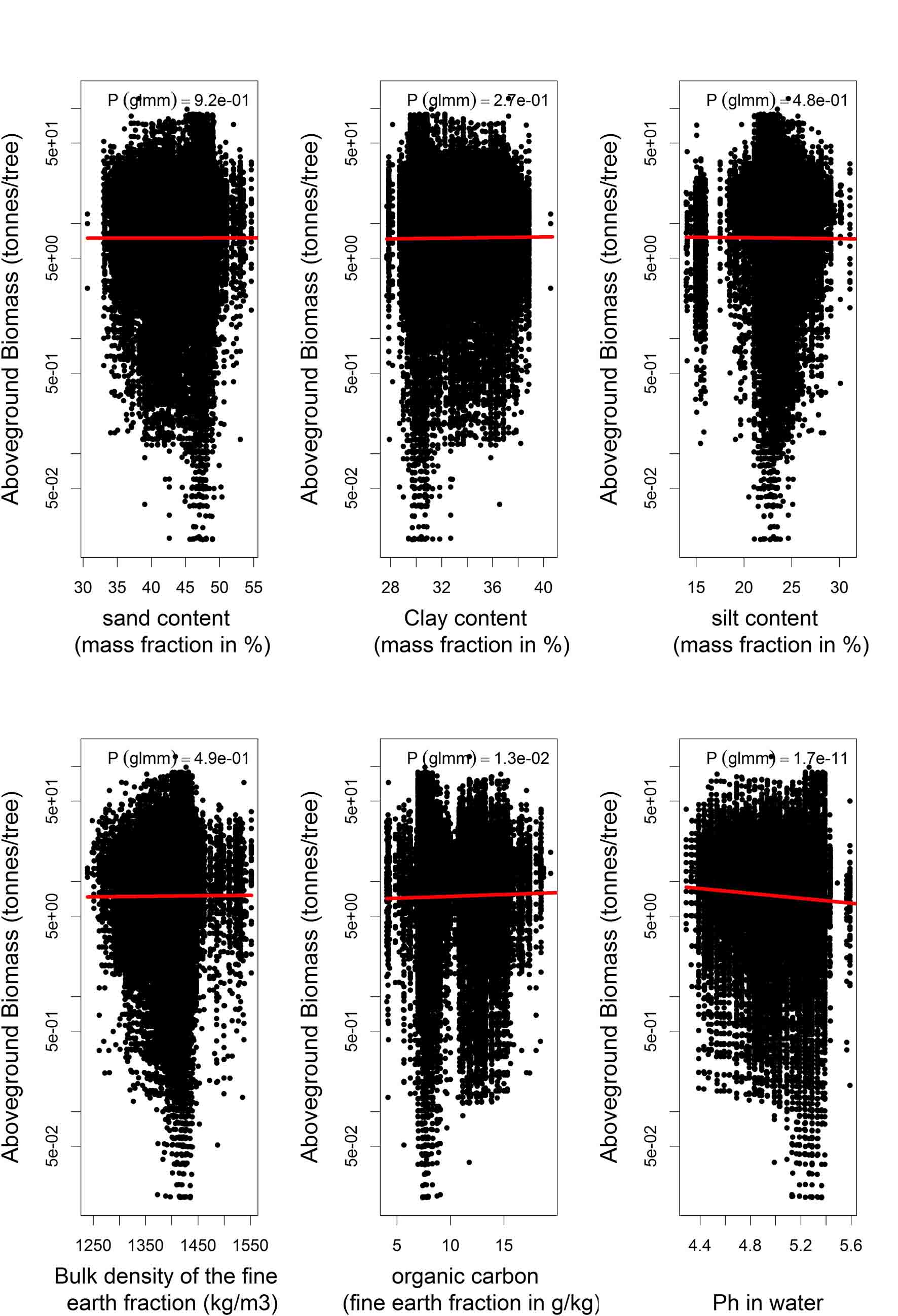


**Figure S10** Relation between estimated AGB of individual Brazil nut trees and soil variables. Please note that the average estimated AGB of all trees in our dataset was 10.25±8.07 tonnes per tree.


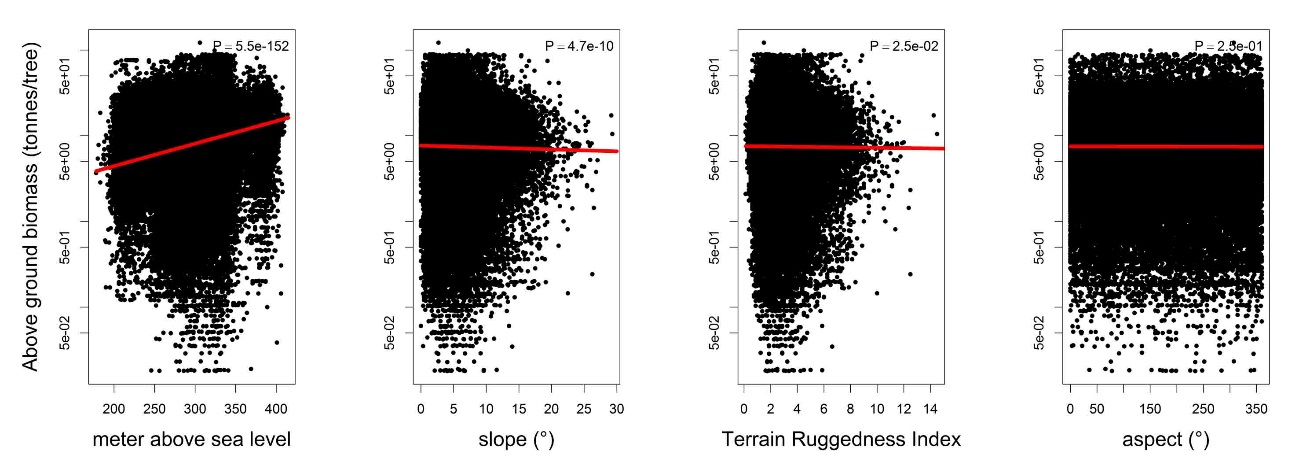


**Figure S11** Relation between estimated AGB of individual Brazil nut trees and terrain variables. Please note that the average estimated AGB of all trees in our dataset was 10.25±8.07 tonnes per tree.


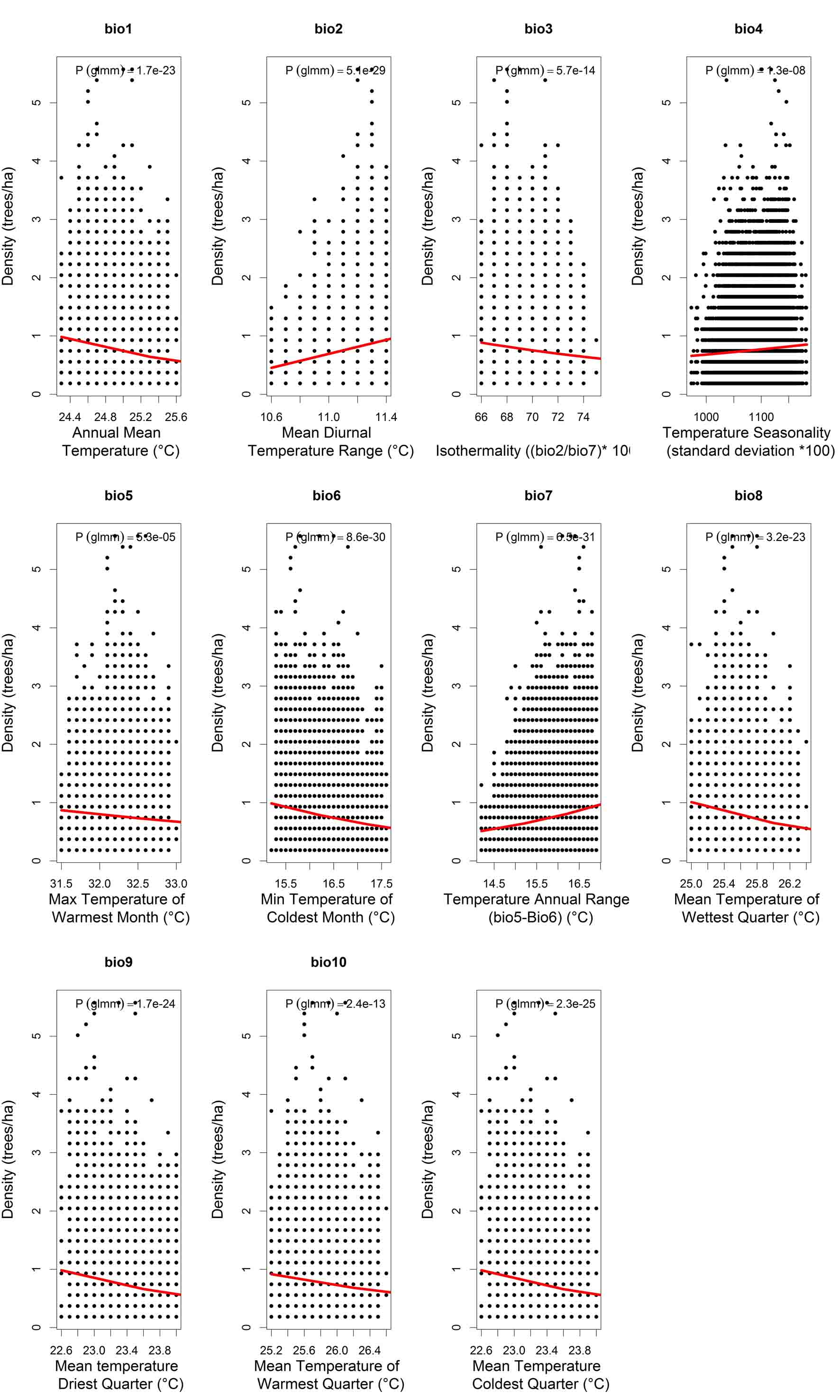


**Figure S12** Relation between Brazil nut tree density and air temperature variables.


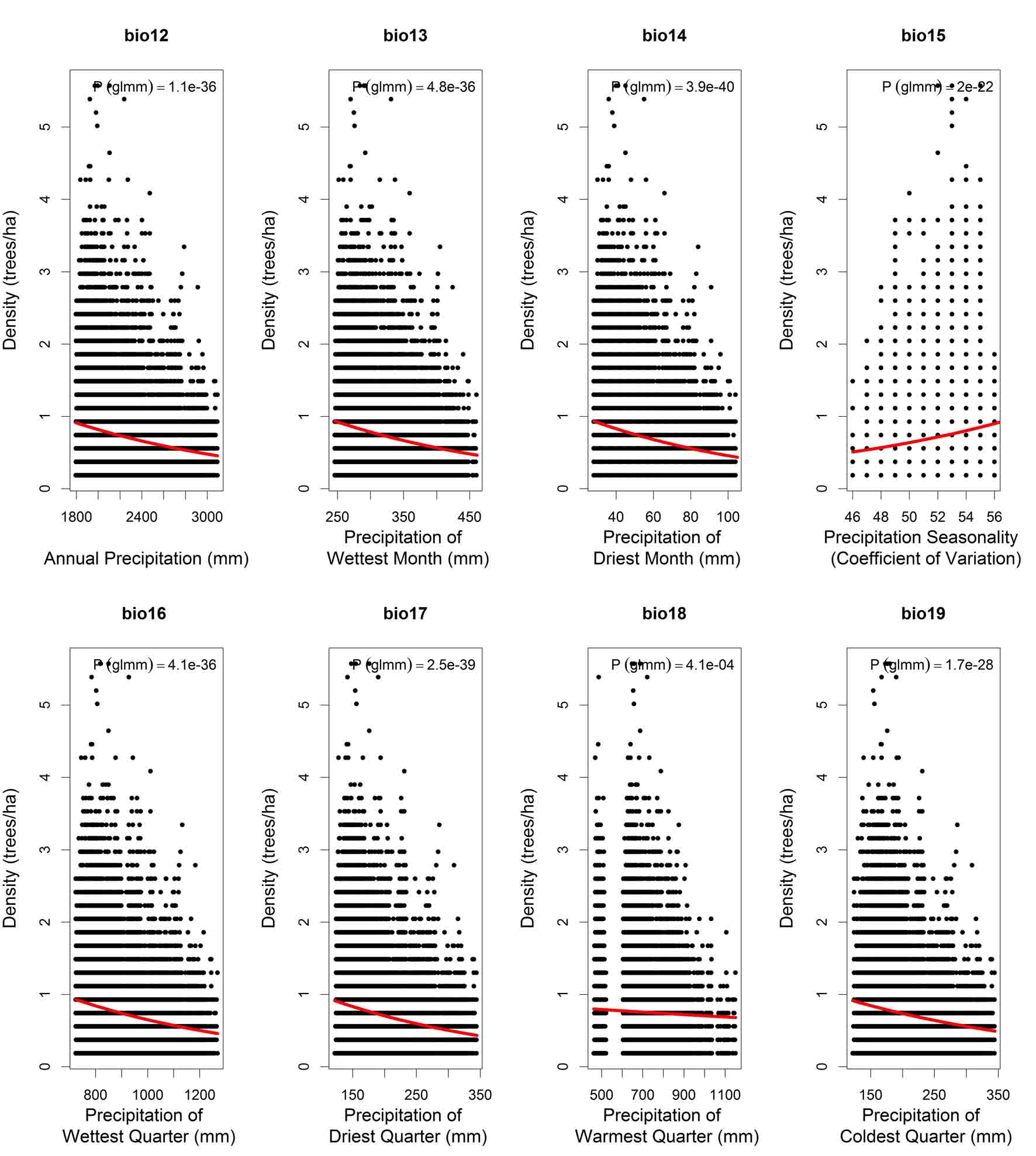


**Figure S13** Relation between Brazil nut tree density and precipitation variables.


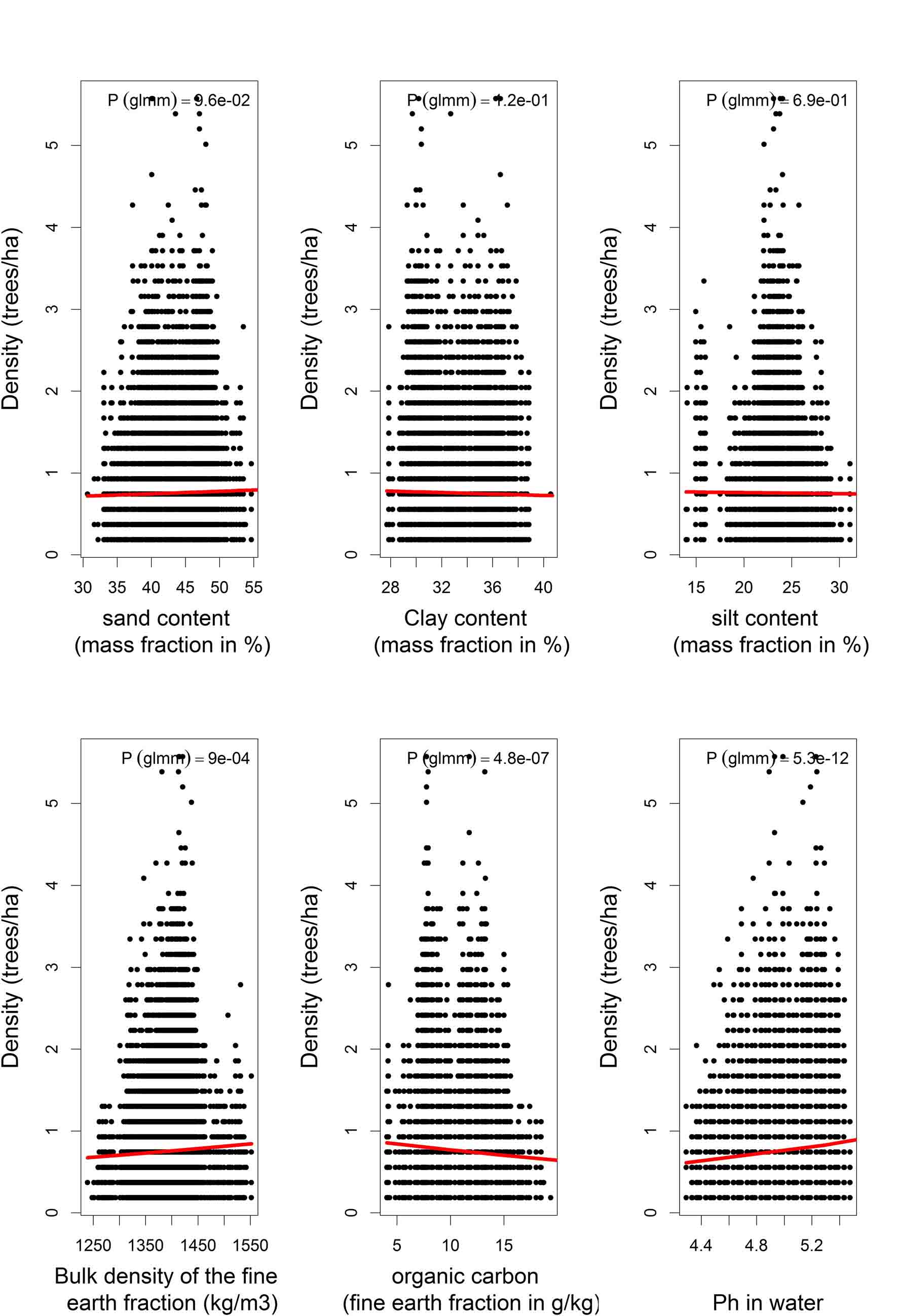


**Figure S14** Relation between Brazil nut tree density and soil variables.


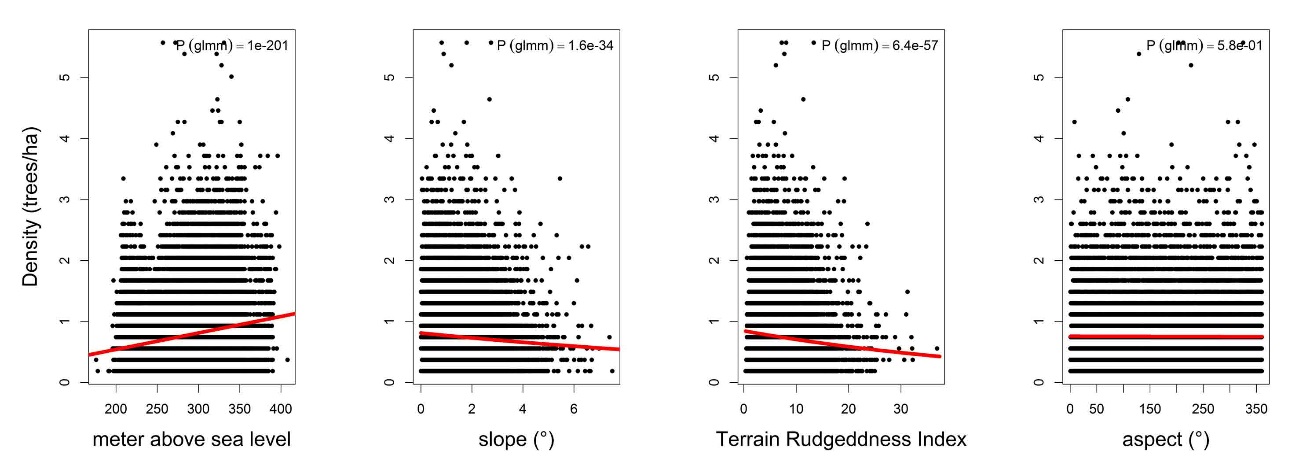


**Figure S15** Relation between Brazil nut tree density and terrain variables.


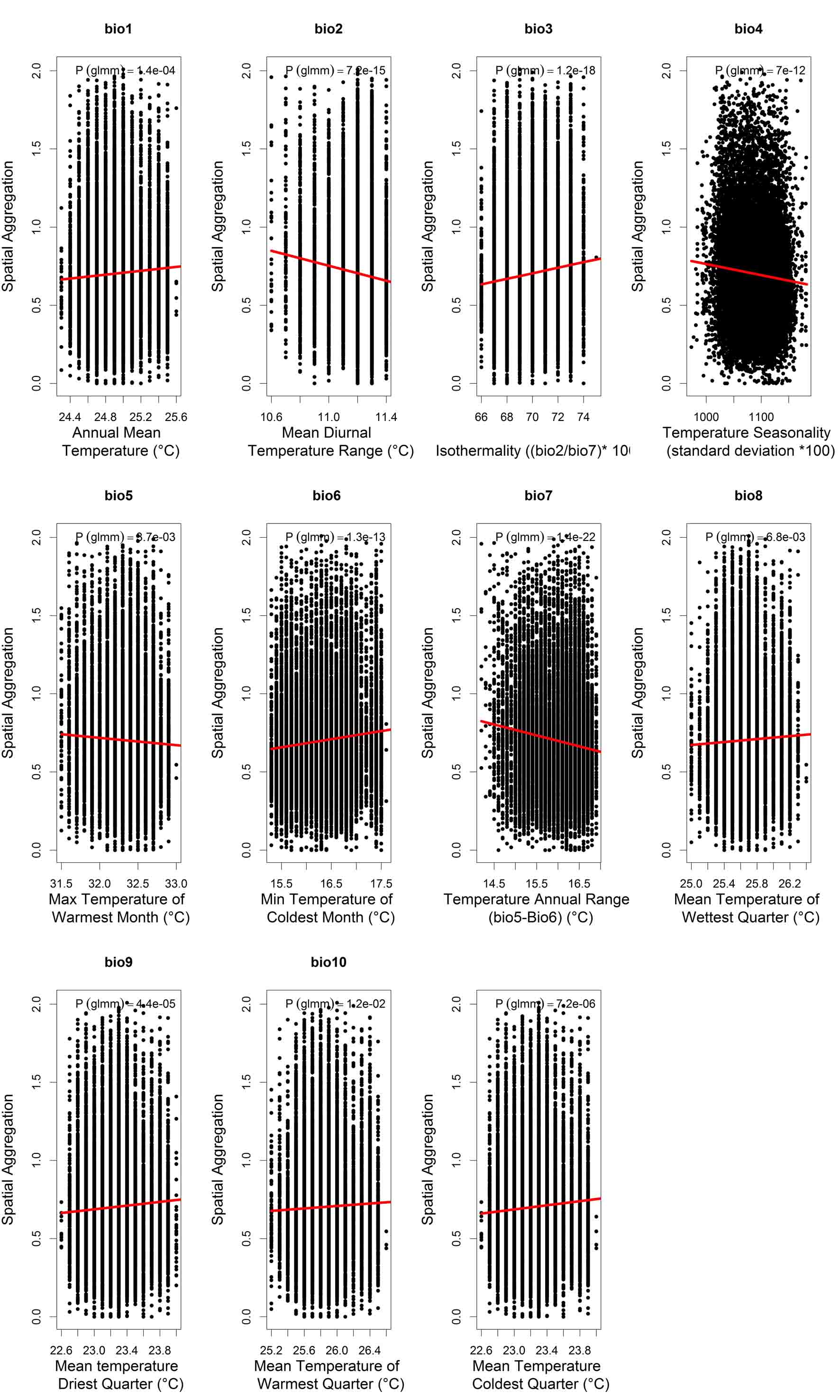


**Figure S16** Relation between spatial aggregation of Brazil nut trees and air temperature variables. A value of 1 is indicative for random patterns, more than 1 for regular (spaced-out) patterns, and less than 1 for aggregated patterns.


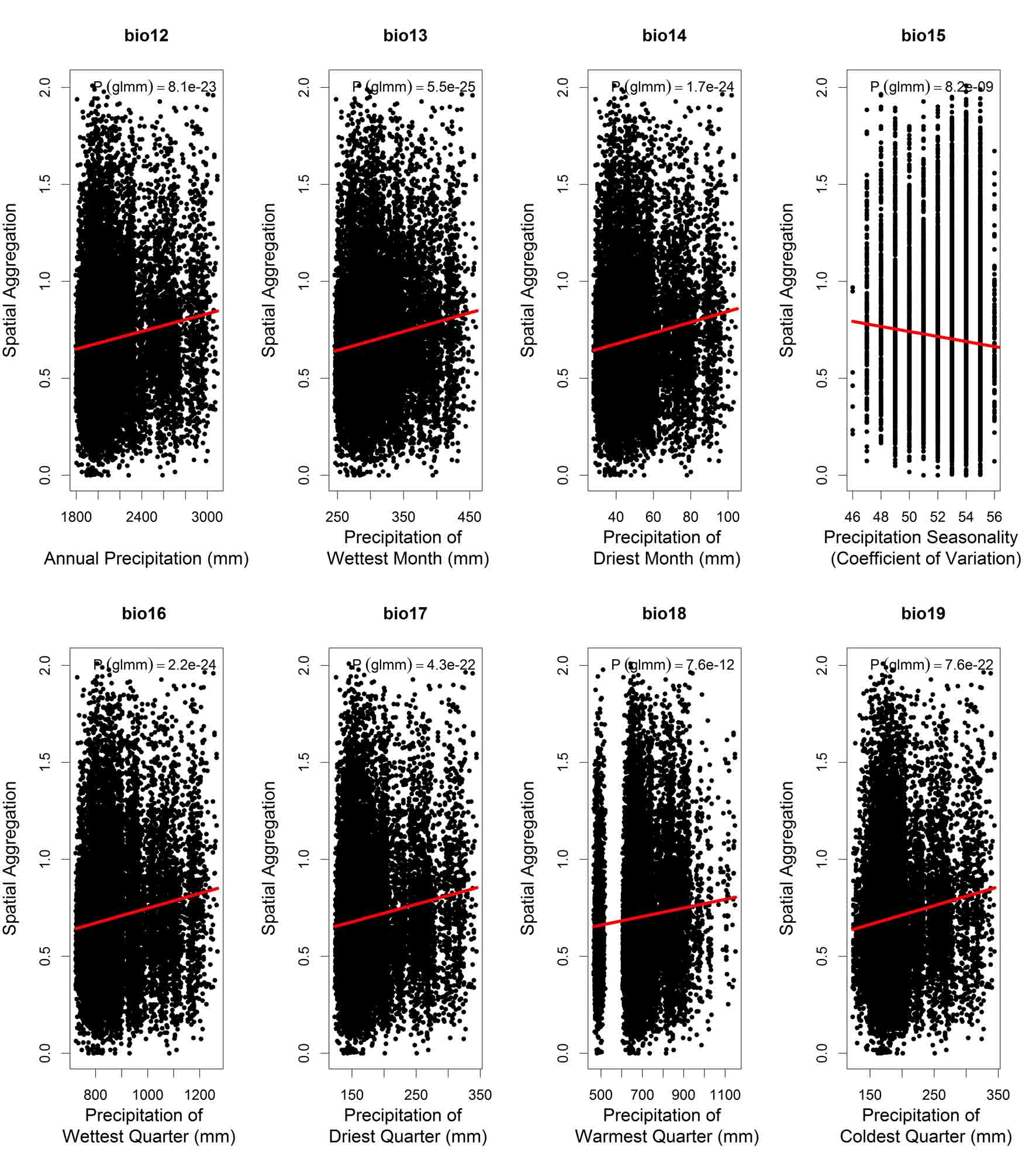


**Figure S17** Relation between spatial aggregation of Brazil nut trees and precipitation variables. A value of 1 is indicative for random patterns, more than 1 for regular (spaced-out) patterns, and less than 1 for aggregated patterns.


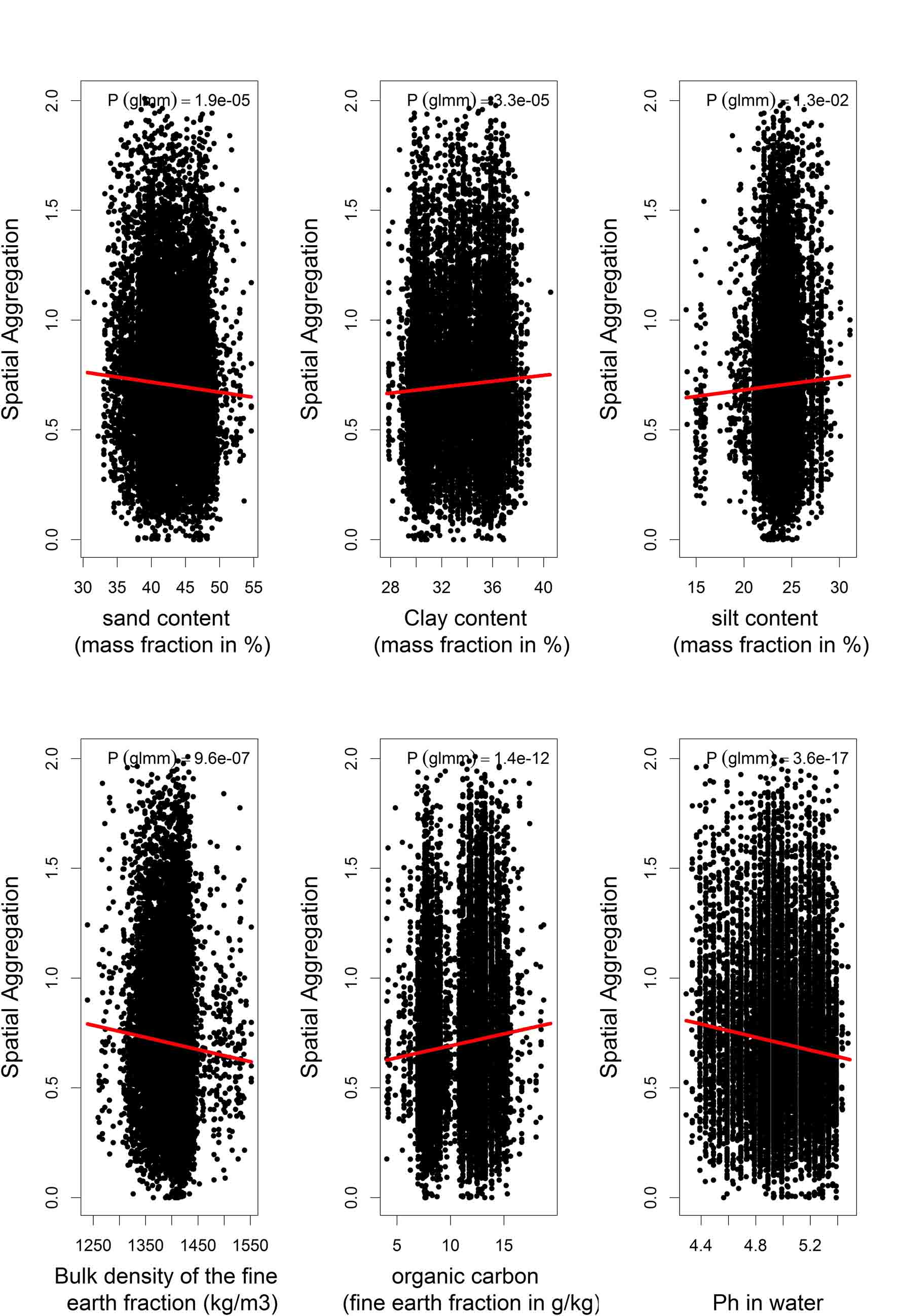


**Figure S18** Relation between spatial aggregation of Brazil nut trees and soil variables. A value of 1 is indicative for random patterns, more than 1 for regular (spaced-out) patterns, and less than 1 for aggregated patterns.


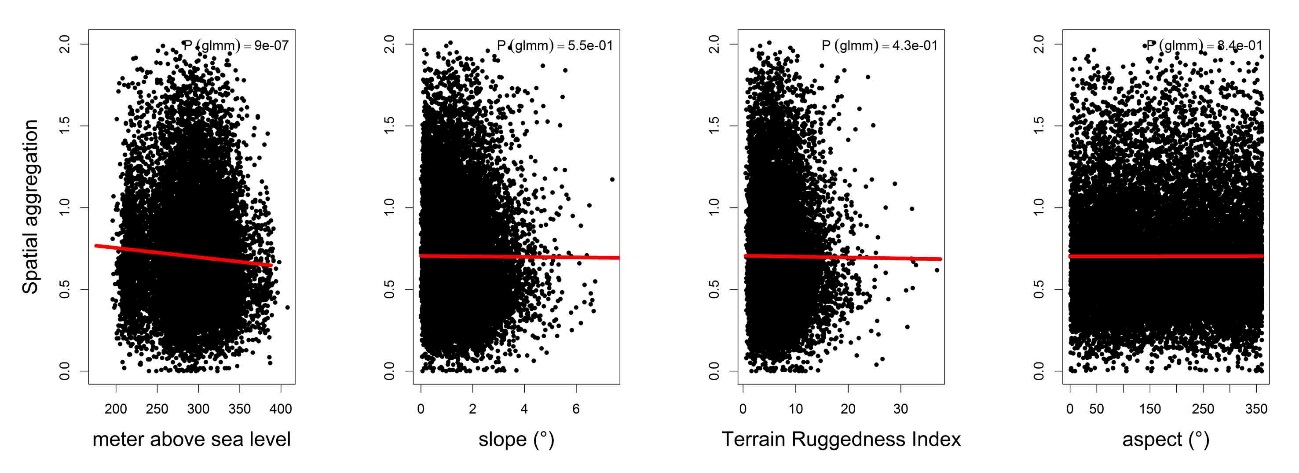


**Figure S19** Relation between spatial aggregation of Brazil nut trees and terrain variables. A value of 1 is indicative for random patterns, more than 1 for regular (spaced-out) patterns, and less than 1 for aggregated patterns.


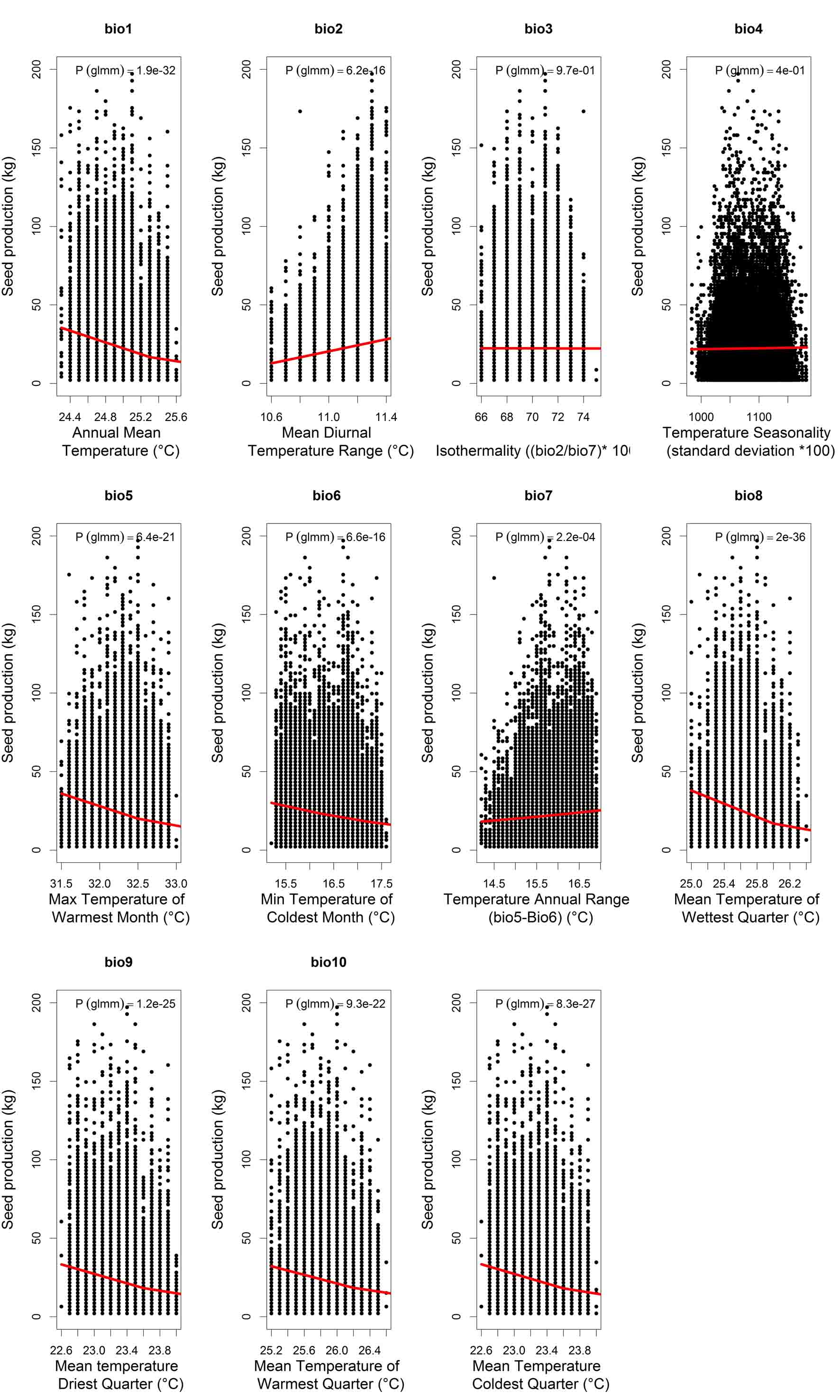


**Figure S20** Relation between estimated seed production of Brazil nut trees per 7.5 arc second grid cells and temperature variables. Seed production values are rescaled to hectare unit.


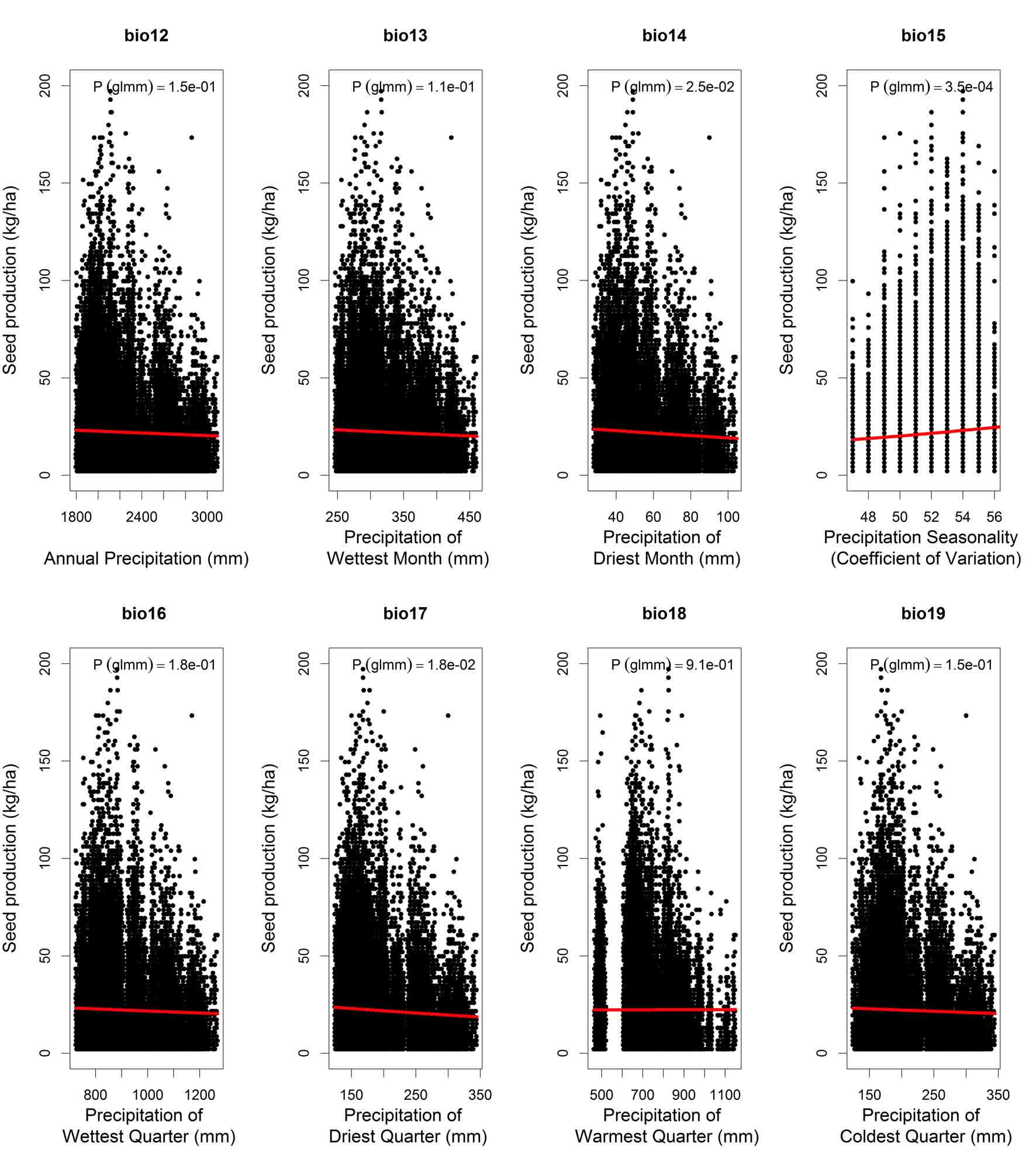


**Figure S21** Relation between estimated seed production of Brazil nut trees per 7.5 arc second grid cells and precipitation variables. Seed production values are rescaled to hectare unit.


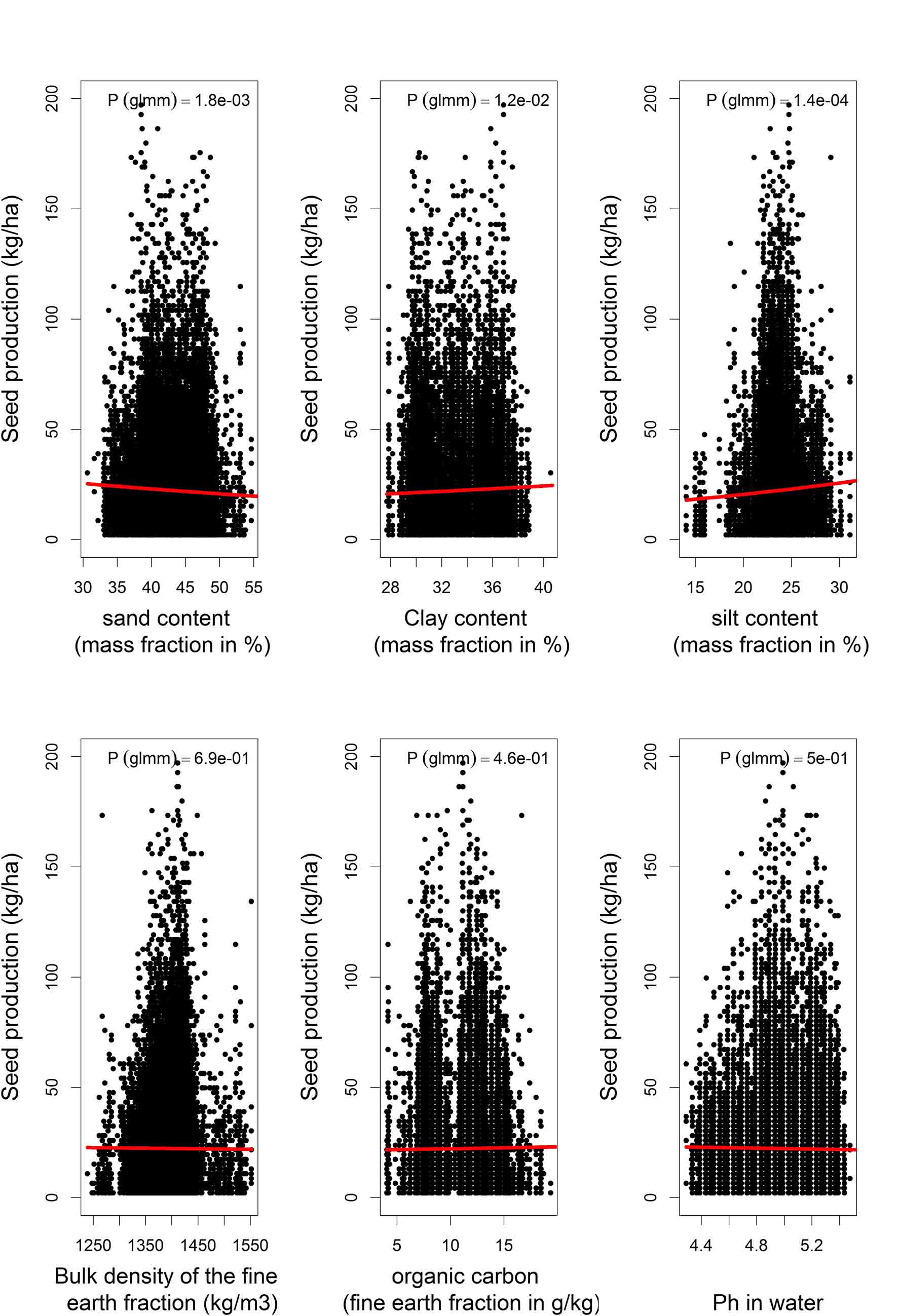


**Figure S22** Relation between estimated seed production of Brazil nut trees per 7.5 arc second grid cells and soil variables. Seed production values are rescaled to hectare unit.


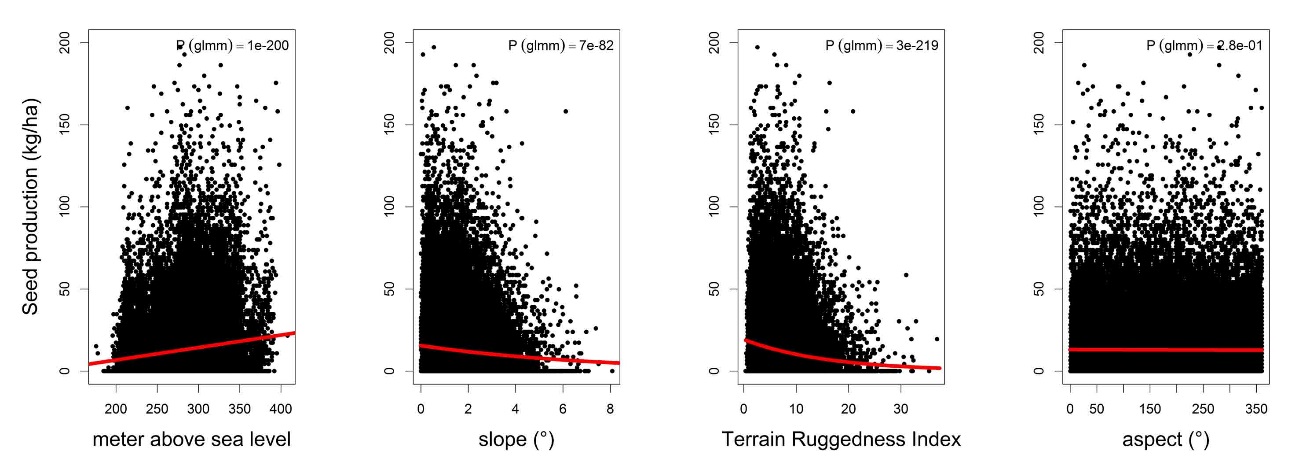


**Figure S23** Relation between estimated seed production of Brazil nut trees per 7.5 arc second grid cells and terrain variables. Seed production values are rescaled to hectare unit.


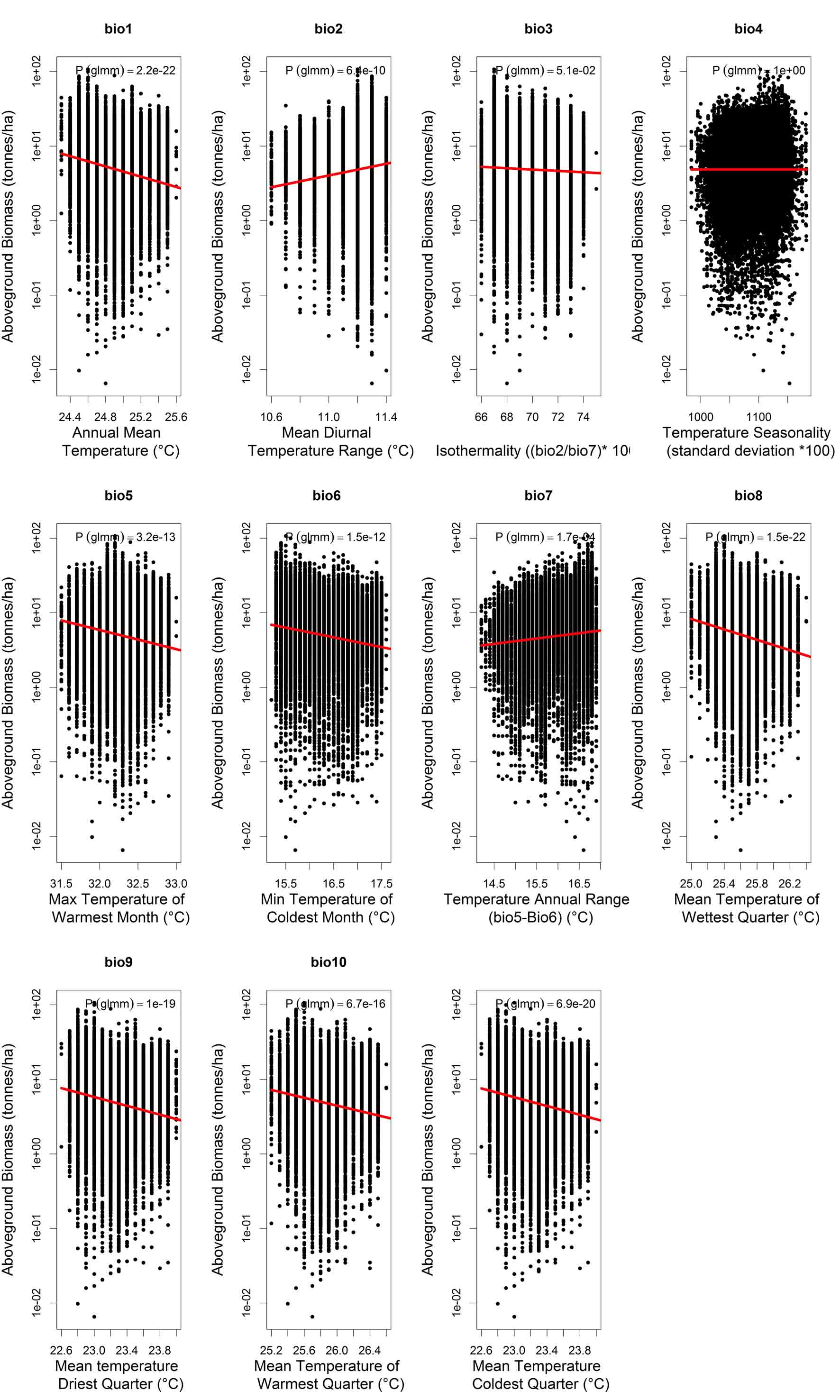


**Figure S24** Relation between estimated AGB of Brazil nut trees per 7.5 arc second grid cells and temperature variables. AGB values are rescaled to hectare unit.


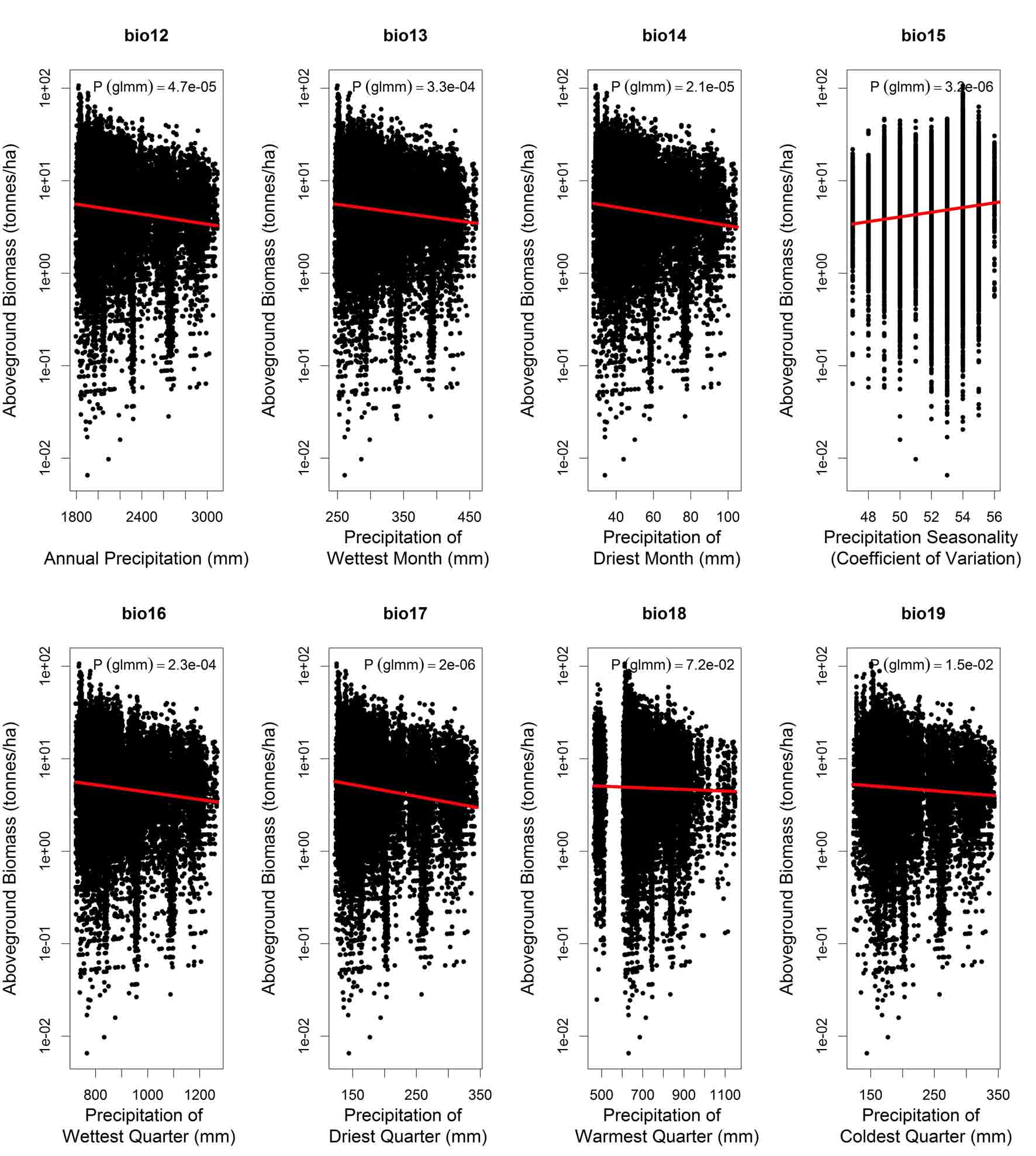


**Figure S25** Relation between estimated AGB of Brazil nut trees per 7.5 arc second grid cells and precipitation variables. AGB values are rescaled to hectare unit.


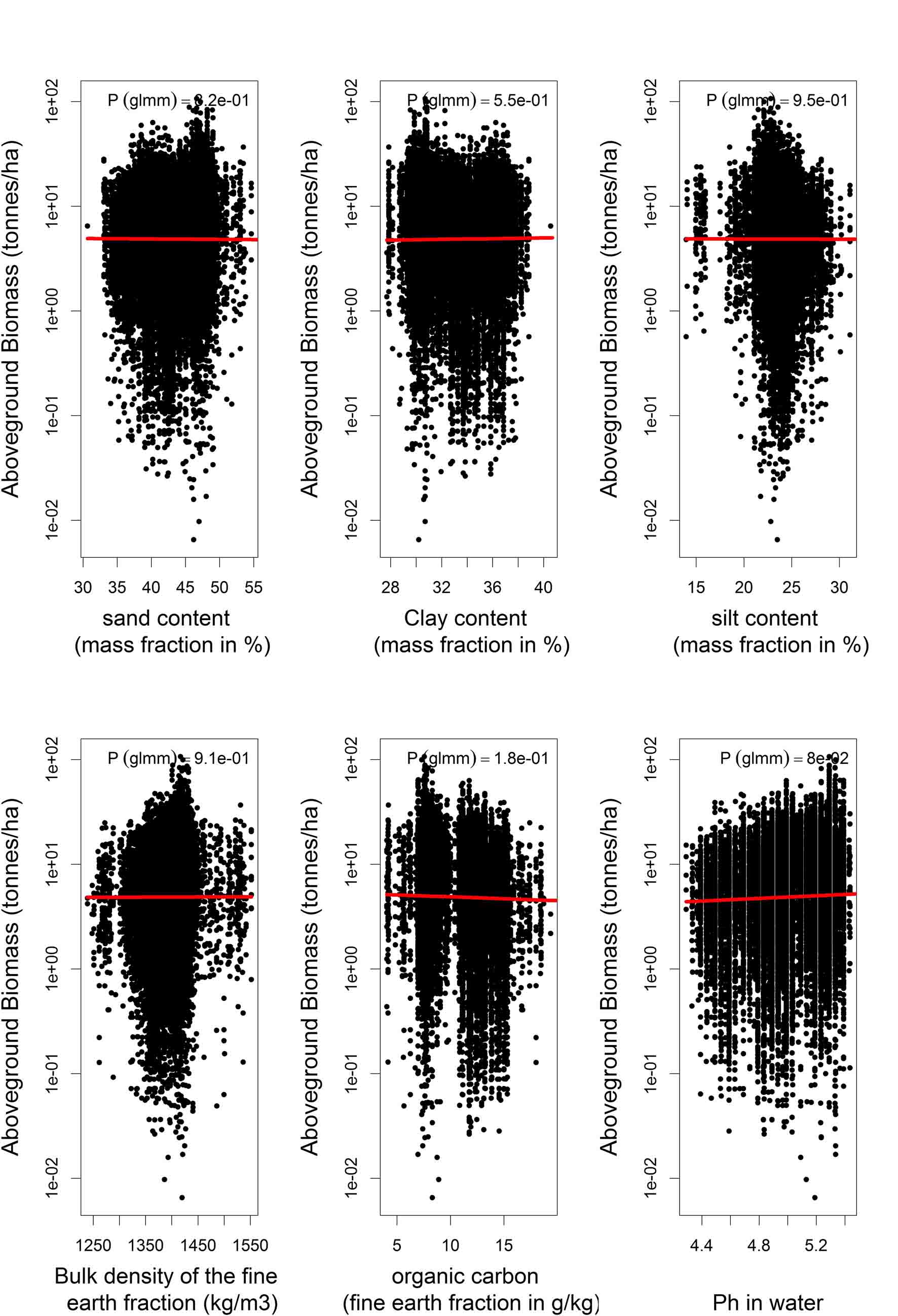


**Figure S26** Relation between estimated AGB of Brazil nut trees per 7.5 arc second grid cells and soil variables. AGB values are rescaled to hectare unit.


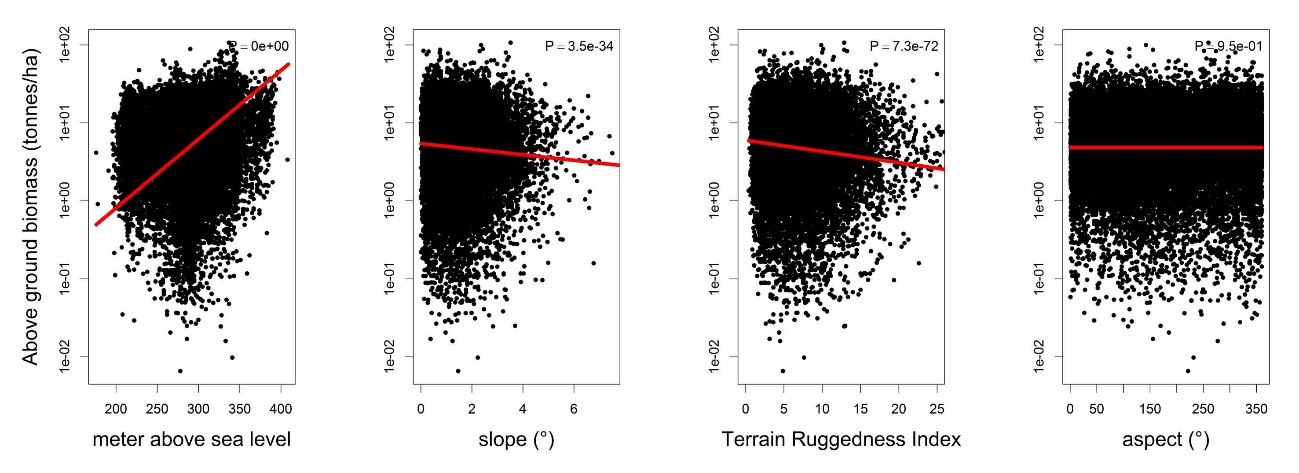


**Figure S27** Relation between estimated AGB of Brazil nut trees per 7.5 arc second grid cells and terrain variables. AGB values are rescaled to hectare unit.
